# Growth Inhibition of Triple-Negative Breast Cancer: The Role of Spatiotemporal Delivery of Neoadjuvant Doxorubicin and Cisplatin

**DOI:** 10.3390/ph14101035

**Published:** 2021-10-12

**Authors:** Dominick Salerno, Stavroula Sofou

**Affiliations:** 1 Chemical and Biomolecular Engineering (ChemBE), Institute for NanoBioTechnology (INBT), Baltimore, MD 21218, USA; dsalern1@jhu.edu; 2 Sidney Kimmel Comprehensive Cancer Center, Cancer Invasion & Metastasis Program, Department of Oncology, Johns Hopkins University, Baltimore, MD 21218, USA

**Keywords:** doxorubicin, cisplatin, combination chemotherapy, tumor spatiotemporal delivery, triple-negative breast cancer, liposomes, lipid nanoparticles

## Abstract

Combinations of platinum-based compounds with doxorubicin in free and/or in liposomal form for improved safety are currently being evaluated in the neoadjuvant setting on patients with advanced triple-negative breast cancer (TNBC). However, TNBC may likely be driven by chemotherapy-resistant cells. Additionally, established TNBC tumors may also exhibit diffusion-limited transport, resulting in heterogeneous intratumoral delivery of the administered therapeutics; this limits therapeutic efficacy in vivo. We studied TNBC cells with variable chemosensitivities, in the absence (on monolayers) and presence (in 3D multicellular spheroids) of transport barriers; we compared the combined killing effect of free doxorubicin and free cisplatin to the killing effect (1) of conventional liposomal forms of the two chemotherapeutics, and (2) of tumor-responsive lipid nanoparticles (NP), specifically engineered to result in more uniform spatiotemporal microdistributions of the agents within solid tumors. This was enabled by the NP properties of interstitial release, cell binding/internalization, and/or adhesion to the tumors’ extracellular matrix. The synergistic cell kill by combinations of the agents (in all forms), compared to the killing effect of each agent alone, was validated on monolayers of cells. Especially for spheroids formed by cells exhibiting resistance to doxorubicin combination treatments with both agents in free and/or in tumor-responsive NP-forms were comparably effective; we not only observed greater inhibition of outgrowth compared to the single agent(s) but also compared to the conventional liposome forms of the combined agents. We correlated this finding to more uniform spatiotemporal microdistributions of agents by the tumor-responsive NP. Our study shows that combinations of NP with properties specifically optimized to improve the spatiotemporal uniformity of the delivery of their corresponding therapeutic cargo can improve treatment efficacy while keeping favorable safety profiles.

## 1. Introduction

Breast cancer is the most commonly diagnosed cancer in women [1]. Approximately 10–15% of all breast cancers are defined as triple-negative breast cancers (TNBC), with known cell surface molecular markers (estrogen, progesterone, and human epidermal growth factor receptor 2 (HER2)) not being highly expressed. TNBC is associated with a poor prognosis, has a high recurrence, and has the lowest 5-year survival rate amongst all breast cancer patients [2]. The lack in tumor selectivity of available treatments (mostly chemotherapies) is partly the reason for current limited efficacy; the heterogeneity of TNBC cells [3], which exhibit a spectrum of sensitivities to (chemo)therapeutic agents, is another. Lastly, the heterogeneous microdistributions of administered chemotherapeutics within the solid TNBC primary and metastatic tumors may be an additional key aspect that reduces efficacy in the clinic since cancer cells that do not get exposed to lethal levels of therapeutic agents may not be killed [4]. 

In advanced (stage II–III) TNBC, neoadjuvant chemotherapy, followed by surgery and by additional chemotherapy, aims to improve the pathological complete response (pCR) in the breast and axilla, which is considered a reliable prognostic factor in terms of recurrence and survival. Some TNBC tumors may likely be driven by chemotherapy-resistant cells that may be the reason behind the limited response to neoadjuvant chemotherapy [3]. Therefore, several ongoing clinical trials evaluate the efficacy of a variety of neoadjuvant treatment schemes to ultimately improve the pCR. These schemes combine therapeutics that act in different ways: either by killing the cancer cells, stopping their division, and/or limiting their ability to spread. The simultaneous use of agents, each acting on a different front, may address the intrinsic heterogeneity of tumor cells comprising the TNBC tumors [3]. 

However, although clinical results with the neoadjuvant combination of doxorubicin (DXR) and cisplatin (CDDP), followed by surgery and then additional chemotherapy, have demonstrated improved pCR, the tolerability profiles of these combinations of chemotherapeutics (in their free form) have been challenging [5,6,7]. As a result, ongoing clinical trials evaluate combinations of these same types of neoadjuvant chemotherapeutics that are either interchangeably replaced with liposomal forms (liposomal doxorubicin such as Doxil, [8]) and/or with different platinum compounds (for example, carboplatin instead of cisplatin). Each of these different forms of chemotherapeutics may exhibit different pharmacokinetics and, importantly, different spatiotemporal microdistributions within tumors, variability in bioavailability (encapsulated vs. free agents), and/or different intrinsic activities against cancer cells (such as cisplatin vs. carboplatin [8]) affecting, therefore, the toxicity and therapeutic efficacy. 

In this study on TNBC cells with variable chemosensitivities, in the absence (on monolayers) and presence (in 3D multicellular spheroids) of transport barriers, in order to evaluate the differences in the efficacy of various delivery forms of these two types of chemotherapeutics, with each form also exhibiting different tolerance profiles [8], we compared (1) the killing effect of free doxorubicin and of free cisplatin to (2) the killing effect of established liposomal forms of the two chemotherapeutics (a liposomal doxorubicin comparable to Doxil and a liposomal cisplatin comparable to Lipoplatin [9]) as well as to (3) the killing effect of tumor-responsive lipid nanoparticles (NP) specifically engineered to result in more uniform spatiotemporal microdistributions of doxorubicin and of cisplatin within solid tumors (responsive DXR-NP and responsive CDDP-NP, respectively).

**Figure 1:**
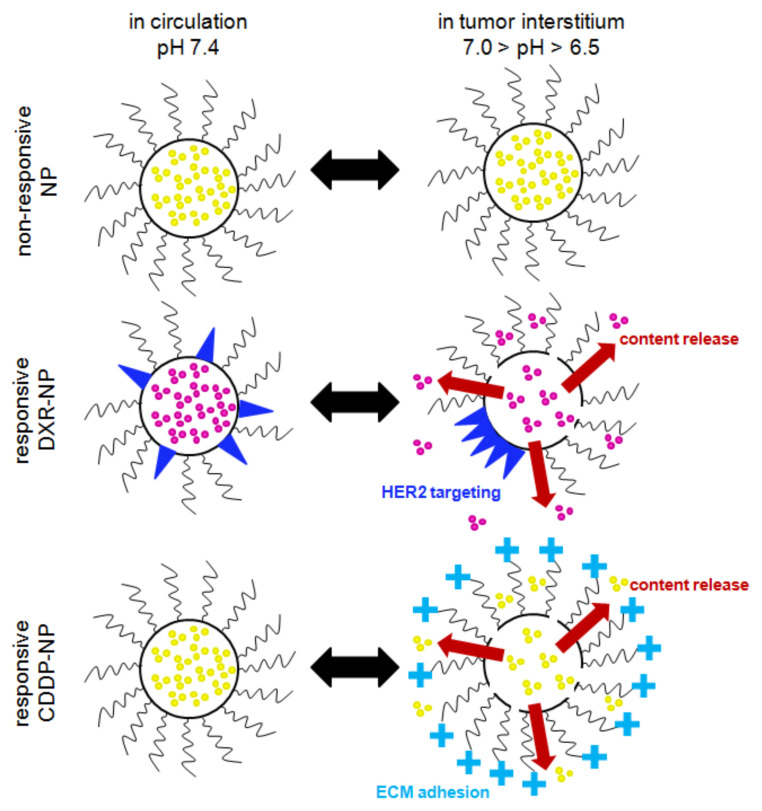
Illustration depicting the properties of NP types. Conventional non-responsive NP (conventional liposomes) retain their contents both in circulation and at the tumor site. Responsive DXR-NP were designed to release their contents only in the acidic tumor interstitium, as well as to form patches of HER2 targeting peptide-ligands (blue triangles) that allow for the targeting of single HER2 receptors on TNBC cells. Responsive CDDP-NP were similarly designed to retain their contents and to exhibit neutral charge in circulation at neutral pH, but when exposed to the decreased pH in the acidic tumor interstitium, to release their contents and gain a positive charge on the free ends of PEG chains, allowing them to adhere to the negatively charged tumor extracellular matrix (ECM) slowing their clearance from tumors. Nanoparticles—NP; cisplatin—CDDP; doxorubicin—DXR; triple-negative breast cancer—TNBC.

The tumor-responsive lipid NP were each engineered with two properties (Figure 1): (a) the property of content release and (b) the property of adhesion/binding to certain targets in the tumors, both designed to be activated by the slightly acidic pH in the tumor interstitium (pH_e_ ~6.7–6.5) [10]; the latter is common in TNBC [11] and is also associated with highly metastatic disease [10,12,13]. NP for both DXR [14] and CDDP [15] were designed to release their therapeutic contents directly in the tumor interstitium in an effort to improve the uniformity of the spatiotemporal profiles of the therapeutic agents within solid tumors. We have previously demonstrated that release in the tumor interstitium of molecular-sized therapeutics, which have significantly higher diffusivities than their nanometer-sized carriers, enabled their deeper penetration in solid tumors, reaching more cancer cells and improving efficacy [16,17]. On both types of responsive NP, the release mechanism was enabled by the formation of phase-separated lipid domains on the membrane comprising the NP. The domain boundaries were tuned to be permeable to the encapsulated agents due to the formation of transient lipid-packing defects that crossed the bilayer [18,19].

For doxorubicin, in addition to the interstitial release, the responsive DXR-NP were also designed to bind to single HER2 receptors followed by cell internalization in an effort to improve the intracellular uptake of DXR in more acidic tumor regions. This second property on doxorubicin-containing carriers was added because doxorubicin exhibits a pK_a_ value of 8.2 [20], which, in the acidic tumor interstitium, may reduce the diffusivity of the free agent (that is released from NP) to passively cross the cell plasma membrane. HER2-mediated uptake of responsive DXR-NP has been shown to increase the intracellular levels of DXR in acidic conditions and to improve cell kill compared to non-internalized NP [14]. The targeting mechanism of NP was enabled by including, in the lipid membrane, lipopeptides targeting the HER2 [14]: during circulation in the blood, the functionalized lipids were shown to be uniformly dispersed on the surface of NP, which were not particularly active towards HER2, and, when in the tumor interstitium, the functionalized lipids preferentially partitioned into phase-separated lipid domains forming ‘patches’ with high local peptide valency, resulting in long binding times of the patch (in a single NP) to a single HER2 receptor followed by cell internalization.

For cisplatin, which efficiently diffuses through cell membranes independent of the extracellular pH, the responsive CDDP-NP were engineered to not become internalized by cancer cells but to adhere to the tumors’ extracellular matrix (ECM) so as to delay their clearance from tumors, therefore releasing more of their chemotherapeutic contents in the tumor interstitium. This, as we have demonstrated, results in delivering more dose at the tumor improving tumor growth inhibition [17]. The NP property of the pH-responsive ECM affinity was enabled by including the titratable moiety dimethyl ammonium propane (DAP) on the free ends of PEG chains that were grafted on the NP surface. The pK_a_ of DAP is approximately 6.58–6.81 [21]; therefore, during circulation in the blood, the surface of these NP maintained electrostatic neutrality with the NP exhibiting identical pharmacokinetics to conventionally PEGylated NP, and when in the tumor interstitium, protonation of DAP resulted in a measurable increase in zeta potential. We demonstrated that the presentation of a cationic charge on an undulating PEG chain significantly reduced the NP interactions with cancer cells, limiting their cell internalization, but it retained measurable adhesion to the tumors’ ECM [16,17]. 

Herein, spheroids, which were utilized as surrogates of the tumor avascular regions, were designed—as we have previously demonstrated—to capture the critical diffusion-limited transport of therapeutics within solid tumors: the trends in efficacy, among various treatments, to inhibit spheroid growth are directly translated on inhibiting tumor growth (and/or delaying metastatic onset) in mouse models bearing the same cancers, as long as the duration times of incubation of spheroids with each of the agents (or formulations thereof) are scaled to their corresponding blood clearance times in vivo [16,17]. In this study, we compared the efficacy to inhibit spheroid growth to the corresponding spatiotemporal microdistributions of each of the agents, in all delivery forms, alone and in combination.

## 2. Results

### 2.1. Nanoparticle Characterization

All NP compositions were prepared reproducibly and exhibited similar drug-to-lipid ratios for both therapeutics (Table 1 and Table 2). Responsive DXR-NP showed significant content release at pH 6.5 (also in Supplemental Figure S1). Additionally, DXR-responsive NP showed significantly greater binding to MDA-MB-231 cells than the non-responsive DXR-NP at the acidic extracellular pH of 6.5 (chosen to correspond to reported values of the pH of the tumor interstitium [10]), validating the use of the HER2-targeting responsive DXR-NP to selectively target low HER2-expressing cancer cells in the acidic tumor microenvironment, as we previously reported [14]. 

The responsive CDDP-NP (Table 2) also showed a significant release of contents under acidic conditions (also in Supplemental Figure S1). Additionally, the zeta potential of the CDDP-responsive NP significantly increased as pH decreased; this was mostly attributed to the protonation of the moiety DAP on the free ends of grafted PEG chains. This cationic charge was previously shown to enable these NP to adhere to the negatively-charged tumor ECM (only under acidic conditions), resulting in slower NP clearance from the tumors [17]. For both agents (Table 1 and Table 2), the non-responsive NP exhibited zeta potential values that remained constant as pH decreased and exhibited, in acidic pH values, significantly less release of contents compared to the corresponding responsive NP and to neutral pH conditions.

### 2.2. Cell Line Characterization

As shown in Table 3 (and in Supplemental Figure S2), free DXR exhibited greater IC_50_ values with lowering the extracellular pH on both cell lines contrary to free CDDP, which exhibits pH-independent diffusion across the cell plasma membrane. The doxorubicin-resistant MDA-MB-231 cell line, DXR-Res-231, was shown to have developed resistance only to free DXR—the IC_50_ value for free DXR increased by more than a factor of 2, while the IC_50_ value for free CDDP remained unchanged when compared to the naïve MDA-MB-231 cell line (Supplemental Figure S3). The DXR-Res-231 cell line maintained the same doubling time and HER2 expression when compared to the naïve MDA-MB-231 cell line, allowing for a direct treatment comparison between the two cell lines. The distribution of HER2 expression of each cell line was verified using FACS (Supplemental Figure S4), and both showed similar fluorescence distributions that correlated with the receptor expression levels measured from the K_D_ experiments.

Table 4 shows the IC_50_ values of both cell lines for DXR and CDDP when delivered by responsive and non-responsive NP. In general, for responsive NP, the IC_50_ values were greater at extracellular pH 7.4 than the IC_50_ values at extracellular pH 6.5, in agreement with their corresponding properties. No significant decrease in cell viability was observed in the non-treated cells due to the acidic extracellular pH throughout the experiment incubation (Supplemental Table S1). In acidic conditions, the responsive NP forms for both agents exhibited significantly lower IC_50_ values than the corresponding non-responsive NP (Table 4 and Supplemental Figures S5 and S6). In particular, for DXR-containing NP at the acidic pH, the lower IC_50_ for the responsive NP was attributed to two factors: (1) the specific binding and internalization of responsive NP by the cells and (2) the release of DXR extracellularly from non-internalized responsive NP (Table 1). For CDDP, in acidic extracellular conditions, the difference in the IC_50_ values between the two NP forms (responsive and non-responsive) increased relative to their difference at neutral pH, due to the greater release of CDDP in acidic conditions, from the responsive NP (as shown on Table 2). For CDDP, both NP forms were not designed to bind to cancer cells. The killing of 50% of the population of DXR-Res-231 cells was not reached by the non-responsive DXR-NP due to limitations of the highest NP concentrations that could be prepared (as shown by the IC_50_ curves in Supplemental Figure S6). NP not containing any chemotherapeutic agents did not have any effect on cell viability (Supplemental Figure S7).

### 2.3. Cell Monolayers—Treatment with Free Agents

#### Justification for Combination of Agents for Synergistic Cytotoxic Effects—The Inhibiting Role of Extracellular Acidity

**Figure 2:**
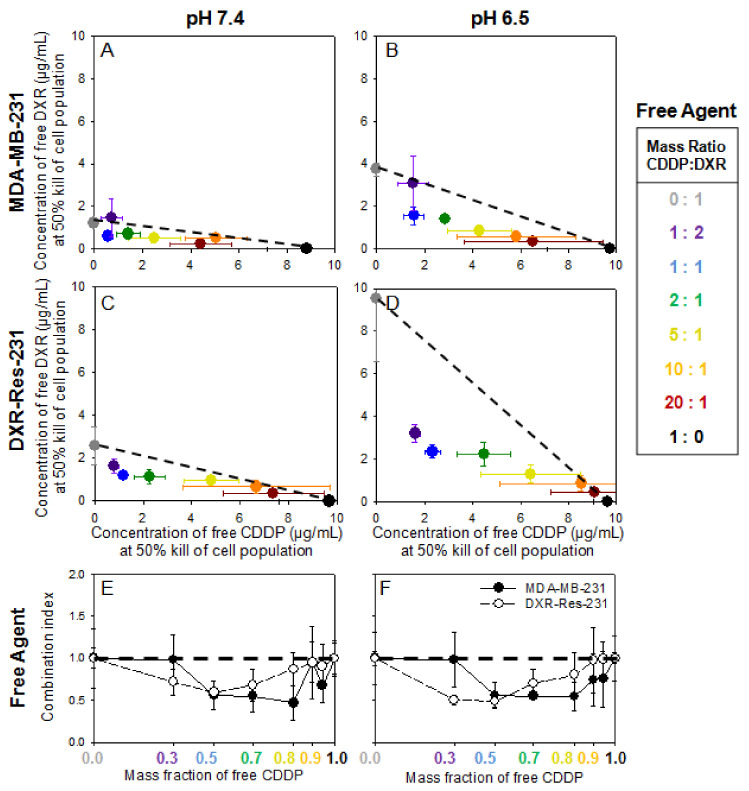
(**A**–**D**). MDA-MB-231 and DXR-Res-231 cell lines. Concentrations of free agents alone and in combination required to kill 50% of cell population (IC_50_), of free DXR and free CDDP introduced alone and in combination on MDA-MB-231 (**A**,**B**) and the DXR-resistant DXR-Res-231 (**C**,**D**) cell lines at pH 7.4 (left column; (**A**,**C**)) and pH 6.5 (right column; (**B**,**D**)) across a range of different CDDP–DXR mass ratios following a 6 h incubation. The dotted lines serve as guide to the eye connecting the single-agent IC_50_ values and illustrate a purely additive relationship between the two agents. If a point falls above (below) this line, it indicates antagonism (synergism) between the two agents. Error bars correspond to standard deviation of *n* = 3 independent measurements (**E**,**F**). Combination Index (CI) of free DXR and free CDDP on MDA-MB-231 (filled symbols) and DXR-Res-231 cell lines (open symbols) at extracellular pH 7.4 (**E**) and pH 6.5 (**F**). The CI is defined as the sum of the normalized IC_50_ values at each ratio CI = ((IC_50_ of free CDDP in combination treatment)/(IC_50_ of free CDDP alone)) + ((IC_50_ of free DXR in combination treatment)/(IC_50_ of free DXR alone)). The horizontal dashed line connects the single-agent combination indices (defined as CI = 1) and illustrates a purely additive relationship between the two agents. If the CI is greater (lower) than 1, it indicates antagonism (synergism) between the two agents. Lines connecting the data points serve as guides to the eye. Error bars correspond to standard deviation of *n* = 3 independent measurements.

Figure 2A–D show the IC_50_ values for free DXR and free CDDP when introduced in combination (at various mass ratios) on both cell lines. At both pH values, the slope of the additivity line (dashed line) increased for the DXR-Res-231 cell line, compared to MDA-MB-231, due to its decreased sensitivity to free DXR. For both cell lines, at pH 6.5, the slope increased relative to the slope at pH 7.4 due to the decreased diffusivity of free DXR across the cell plasma membrane in acidic conditions, as we have previously shown [22]. Across all ratios of agents for both cell lines and conditions, an additive or synergistic relationship was observed (data points were on or below the dashed line), verifying that these two therapeutics could be good candidates when administered in combination. The plots in Figure 2 are shown in terms of the IC_50_ values for each agent, when in combination, so as to illustrate the effect of pH, as well as to compare IC_50_ values between different forms of each agent (free agent, in responsive NP, and/or in non-responsive NP; vide infra). To alternatively demonstrate the synergistic/antagonistic effects of the combination treatment, the normalized IC_50_ values for each agent, when in combination, with respect to the IC_50_ value for each agent as a single treatment, are shown in the Supporting Information (Table S2 and Supplemental Figure S8).

The additivity and/or synergy was further observed when comparing the combination index (CI) (shown in Figure 2E,F). The CI is defined as the sum of the normalized IC_50_ values for each mass ratio of the two agents (CI = ((IC_50_ of free CDDP in combination treatment)/(IC_50_ of free CDDP alone)) + ((IC_50_ of free DXR in combination treatment)/(IC_50_ of free DXR alone))). Values of CI equal to unity indicate an additive response, greater than unity an antagonistic response, and lower than unity a synergistic response (numerical values are shown in Supplemental Table S3) [23]. At both extracellular pH values studied, for MDA-MB-231 cells (closed symbols), the best response was seen when a small amount of free DXR was combined with free CDDP (shown at 0.8 mass fraction of free CDDP). However, for the DXR-Res-231 cells (open symbols), an expected shift towards greater mass ratios of free DXR (≥0.3) was required for killing 50% of the cell population due to the decreased sensitivity of the cell line to free DXR.

### 2.4. Cell Monolayers—Treatment with Agents in NP Forms

#### Activation of Responsive-NP Properties in the Acidic Extracellular Environment Improves Efficacy

**Figure 3:**
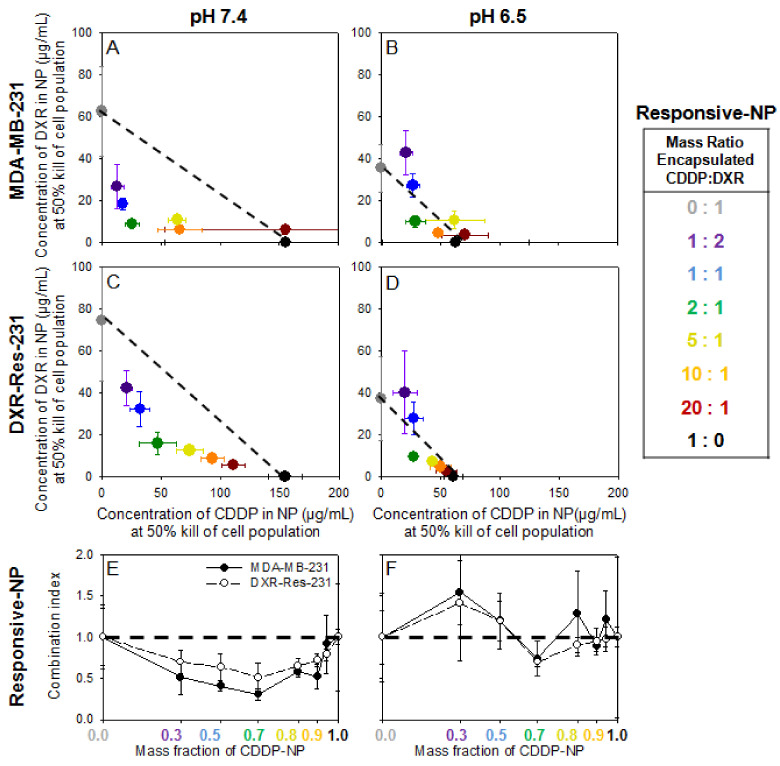
(**A**–**D**). MDA-MB-231 and DXR-Res-231 cell lines. Concentrations of agents delivered by responsive NP alone and in combination required to kill 50% of cell population (IC_50_), of responsive DXR-NP and of responsive CDDP-NP given in combination on MDA-MB-231 (**A**,**B**) and DXR-Res-231 (**C**,**D**) cell lines at pH 7.4 (left column; (**A**,**C**)) and pH 6.5 (right column; (**B**,**D**)) across a range of different encapsulated CDDP–DXR mass ratios following a 6 h incubation. The dotted lines serve as guide to the eye connecting the single-agent IC_50_ values and illustrate a purely additive relationship between the two therapeutics. If a point falls above (below) this line, it indicates antagonism (synergism) between the two agents. Error bars correspond to standard deviation of *n* = 3 independent measurements. (**E**,**F**) Combination Index (CI) of DXR- and CDDP-responsive NP on MDA-MB-231 (filled symbols) and DXR-Res-231 (open symbols) cell lines. The CI is defined as the sum of the normalized IC_50_ values at each ratio CI = ((IC_50_ of responsive CDDP-NP in combination treatment)/(IC_50_ of responsive CDDP-NP alone)) + ((IC_50_ of responsive DXR-NP in combination treatment)/(IC_50_ of responsive DXR-NP alone)). The horizontal dashed line connects the single-agent combination indices (defined as CI = 1) and illustrates a purely additive relationship between the two agents. If the CI is greater (lower) than 1, it indicates antagonism (synergism) between the two agents. Lines connecting the data points serve as guides to the eye. Error bars correspond to the standard deviation of *n* = 3 independent.

Figure 3A–D show the IC_50_ values for each of the agents in responsive-NP forms when introduced alone and in combination on both cell lines. The additive and/or synergistic relationship that was observed with the free agents (Figure 2) was maintained at all mass fractions in neutral extracellular pH (Figure 3A,C) and with several mass ratios in acidic conditions (Figure 3B,D) (individual values and normalized plots seen in Supplemental Tables S4 and S5 and Supplemental Figure S9). For both cell lines, at acidic conditions, the concentrations of formulated agents in responsive NP that were required to kill 50% of the cell population were lower than the concentrations of agents in the same nanoparticles at neutral pH. This was due to the activation of the release and binding/internalization properties on the responsive NP in the acidic conditions. The additive/synergistic relationship was further observed in the CI plots (Figure 3E,F) albeit with larger errors at pH 6.5. Since a significant fraction of agents was still encapsulated in NP even in the acidic pH (see Table 2 and Table 3), the actual concentrations of agents (that induced 50% cell kill) when delivered by responsive NP was greater than the corresponding values of the agents in free form (Figure 2). 

The CI values in Figure 3E,F exhibited relatively lower synergy in the DXR-Res-231 cell population compared to the parent cell line and also compared to the synergistic effect of the combined free agents (Figure 2E,F). In Figure 3, the chemotherapeutic agents were formulated in responsive NP. Therefore, the studies collectively captured different processes that affected the transport/trafficking of each of the chemotherapeutics into the cells, including the release of agents from nanoparticles and the binding/internalization of some of the nanoparticles by the cancer cells. These measurements were performed in the absence of diffusion-limited transport (i.e., not in 3D spheroids but on cell monolayers), which was the main rationale for the design of each of these nanoparticles. The effect of the treatment in 3D cell cultures is shown below.

**Figure 4:**
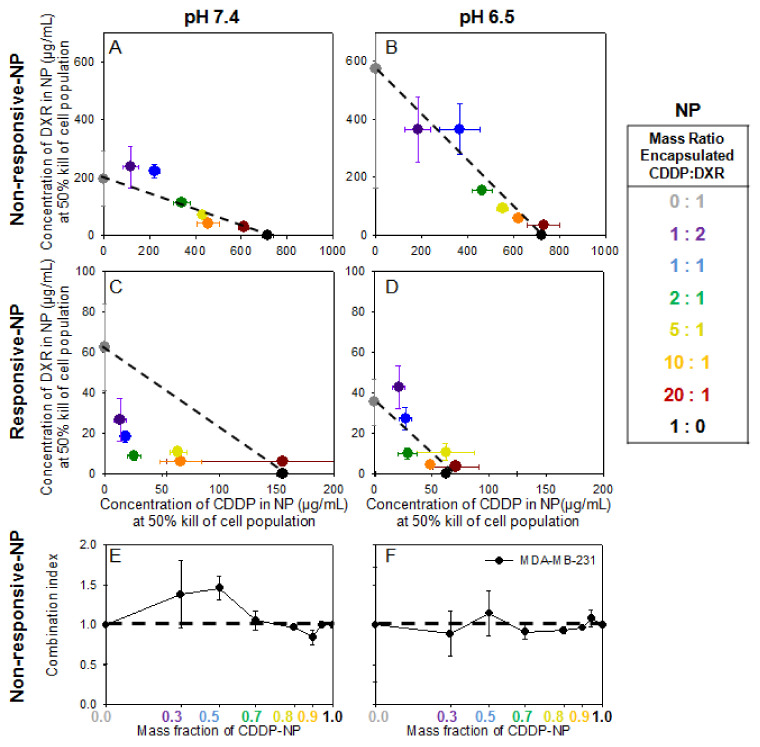
(**A**–**D**). MDA-MB-231 cell line. Concentrations of agents delivered by non-responsive NP compared to the concentrations of same agents delivered by responsive NP, alone and in combination, required to kill 50% of cell population (IC_50_). DXR and CDDP delivered by non-responsive NP (**A**,**B**), and DXR and CDDP delivered by responsive NP (**C**,**D**) given in combination on the MDA-MB-231 cell line at pH 7.4 (left column; (**A**,**C**)) and pH 6.5 (right column; (**B**,**D**)) across a range of different mass ratios of encapsulated CDDP–DXR. The dotted lines serve as guide to the eye connecting the single drug IC_50_ values and illustrate a purely additive relationship between the two therapeutics. If a point falls above (below) this line, it indicates antagonism (synergism) between the two agents. Error bars correspond to standard deviation of *n* = 3 independent measurements. (**E**,**F**). Combination Index (CI) of DXR- and CDDP-non-responsive NP on MDA-MB-231 cell line. The CI is defined as the sum of the normalized IC_50_ values at each ratio CI = ((IC_50_ of non-responsive CDDP-NP in combination treatment)/(IC_50_ of non-responsive CDDP-NP alone)) + ((IC_50_ of non-responsive DXR-NP in combination treatment)/(IC_50_ of non-responsive DXR-NP alone)). The horizontal dashed line connects the single-agent combination indices (defined as CI = 1) and illustrates a purely additive relationship between the two agents. If the CI is greater (lower) than 1, it indicates antagonism (synergism) between the two agents. Lines connecting the data points serve as guides to the eye. Error bars correspond to standard deviation of *n* = 2 independent measurements.

In Figure 4, on MDA-MB-231 cells, both agents, when delivered by non-responsive NP, required an even greater increase in concentrations to induce 50% cell kill when compared to the concentrations of the same agents when delivered by responsive NP (individual values and normalized plots are shown in Supplemental Table S6 and Supplemental Figure S10). This result was attributed to the lower bioavailability of agents since the non-responsive NP were not designed to significantly release their therapeutic contents and/or to actively become internalized by cells. Regardless of delivery carrier, DXR and CDDP generally maintained an additive/synergistic effectiveness when introduced in combination compared to either agent alone. The only exception was certain combinations of the two agents when delivered by non-responsive NP forms at neutral pH (Figure 4E). The concentrations of agents in non-responsive NP forms against DXR-Res-231 are not shown because they were not high enough to result in killing of 50% of the DXR-Res-231 cell population even when combined (data not shown).

### 2.5. Spheroid Characterization and Treatment

Spheroids were used as surrogates of the avascular regions of solid tumors [16,17,24]. Spheroids, which were formed by each of the cell lines, developed interstitial pH profiles that ranged from 6.5 at the spheroid center to around 7.4 at the spheroid periphery (Supplemental Figure S11). These values were comparable to the tumor extracellular pH values that were measured in vivo [10,16] and were within the range of pH values that activated the properties of binding/adhesion and release on responsive NP (Table 1 and Table 2).

Spheroids were treated with each agent in free or in NP-encapsulated form and were exposed to the various agent forms for incubation times relevant to their corresponding blood circulation times (vide supra). When agents were introduced in combination, the mass ratio of 1:1 was used to replicate the combined treatments in clinical studies [5]. Additionally, a lower concentration of the free agents was also studied, 4.5 µg/mL, to represent the lower maximum tolerated dose (MTD) of free agents when compared to the MTD of their corresponding NP formulations [25,26].

**Figure 5:**
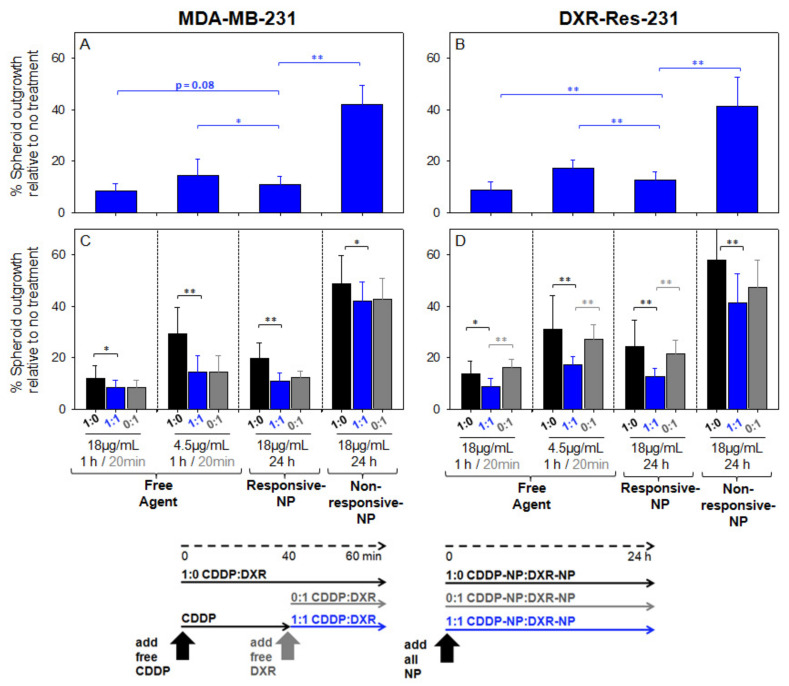
Extent of inhibiting spheroid outgrowth by agents in combination and as a single treatment. Spheroids formed by MDA-MB-231 cells (**A**) and by the doxorubicin-resistant DXR-Res-231 cells (**B**) were treated with 18 or 4.5 µg/mL CDDP combined with 18 or 4.5 µg/mL DXR (1:1, blue bars), respectively. The spheroid outgrowth by the combined treatments was compared to the outgrowth by the corresponding single treatments (**C**,**D**) of 18 or 4.5 µg/mL CDDP (1:0, black bars) and/or 18 or 4.5 µg/mL DXR (0:1, grey bars), respectively, in free and/or NP-form as indicated on the plot. Arrows indicate the treatment schedule: spheroids were incubated with free CDDP for one hour, with free DXR for 20 min, and with all NP forms for 24 h, to model their representative blood clearance kinetics as discussed in the main text. Error bars correspond to standard deviation of *n* = 3 independent measurements (*n* = 6 spheroids per measurement). * indicates *p*-values < 0.05, ** *p*-values < 0.01.

**Figure 6:**
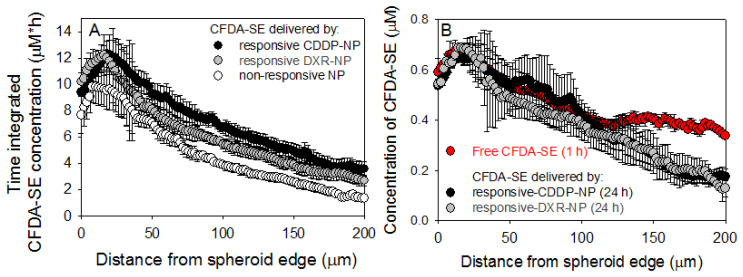
(**A**) Spatiotemporal profiles of the fluorescent drug surrogate CFDA-SE delivered by different NP forms. CFDA-SE was treated as a surrogate of CDDP and of DXR and was loaded in the corresponding responsive and non-responsive NP; compositions for responsive CDDP-NP (black symbols), for responsive DXR-NP (grey symbols), and for non-responsive NP (white symbols). The time-integrated radial concentrations were calculated using the trapezoid rule that integrated the radial spatial microdistributions shown in Supplemental Figures S12 and S13. Error bars correspond to the standard deviation of *n* = 3 spheroids per sampled time point. (**B**) Snapshots of the spatial microdistributions of the fluorescent drug surrogate CFDA-SE in spheroids in free and/or in NP form. Microdistributions of CFDA-SE (used as a surrogate for CDDP and for DXR) in different forms (free and/or in NP) at the relevant end time points of incubation for each form that was scaled to the agents’ blood circulation times. Error bars correspond to standard deviation of *n* = 3 spheroids.

The extent of outgrowth inhibition of spheroids was used as an indication of inhibition of tumor growth and/or recurrence [16]. When treated with the combined agents, the outgrowth of spheroids, formed by MDA-MB-231 cells (Figure 5A) and by DXR-Res-231 cells (Figure 5B), was more effectively inhibited by the free agents and by the responsive NP. The combined agents in non-responsive-NP forms exhibited markedly limited efficacy. This response was consistent with the time-integrated concentrations (radial AUC within the spheroids, AUC_r_) of the therapeutic agent surrogate forms that are shown in Figure 6A (individual uptake and clearance spatiotemporal microdistributions used for integration are shown in Supplemental Figures S12 and S13). The non-responsive-NP form resulted in the lowest AUC_r_ values along the spheroid radius, and both responsive-NP forms increased the AUC_r_ at all radial values.

Particularly in spheroids formed by the DXR-resistant cell line (DXR-Res-231) (Figure 5D), the combined agent treatment exhibited greater outgrowth inhibition by either agent when each was introduced alone, justifying the choice of this particular agent combination for the resistant TNBC tumors.

The agents in their free form were generally more effective in inhibiting spheroid outgrowth compared to their non-responsive-NP form. This result was expected given the higher diffusivities of the free agents and, therefore, their greater penetration within the spheroid interstitium (also shown in Figure 6B by the red symbols). 

## 3. Discussion

Neoadjuvant chemotherapy with combined doxorubicin (DXR) and cisplatin (CDDP)—or other platinum compounds—is currently evaluated in clinical trials for patients with advanced, chemoresistant TNBC. While many patients respond well to neoadjuvant chemotherapy, approximately 30–50% develop chemoresistance, highlighting the importance of developing new treatments to increase overall survival [27,28]. The heterogeneity of TNBC, which is known to exhibit a spectrum of unique biologies [3], is the reason for combining chemotherapeutics acting on different molecular mechanisms; the combinations of different agents are aimed to collectively eradicate the heterogeneous cancer cell populations. Although still under clinical investigation, the synergistic cell kill by combinations of free DXR and free CDDP, compared to the killing effect of each agent alone, was confirmed in this study on monolayers of cells with different chemosensitivities in an effort to mimic TNBC’s biological heterogeneity (Figure 2). Additionally, especially on TNBC spheroids that were formed by cells exhibiting resistance to DXR, the simultaneous treatment with both agents (in free and/or in NP forms) demonstrated greater inhibition of spheroid outgrowth compared to the inhibition of outgrowth when spheroids were treated only by one of the agents (in either free or NP forms).

Importantly, the potentially challenging safety profiles of chemotherapeutics in free form, alone and/or in combination [29], have motivated the use of liposomal forms of the same agents in the clinic. In this study, we validated the choice of using, in combination, lipid nanoparticle (NP) forms of the two agents: on TNBC cells on monolayers, combined NP forms of the two agents exhibited both additive and/or synergistic killing effects, compared to the NP form(s) of each agent alone (Figure 3 and Figure 4). However, as expected, the absolute efficacy (per agent concentration/mass, in vitro) of agents in NP forms was lower compared to the efficacy of free agents due to the reduced bioavailability of NP-encapsulated agents. This reduced killing effect of agents when in NP forms, compared to the killing effect of their free form(s), has also been reported in animal studies [30,31,32].

With this in mind, we designed environmentally responsive NP, designated as responsive NP, that essentially increased the bioavailability of DXR and of CDDP selectively at the tumor sites; the increased bioavailability was enabled by active release of agents from the NP and was activated at slightly acidic pH values of the interstitium of TNBC tumors. On TNBC cells on monolayers, the improved absolute efficacy of agents delivered by these responsive NP, both alone and in combination, was demonstrated by the significantly lower concentrations of agents in responsive NP forms (3- to 15-times lower), compared to agents in conventional NP forms, that were required to kill the same fraction of TNBC cells (Figure 4). 

Additionally, independent of the bioavailability of delivered chemotherapeutics, which was shown to be improved by the responsive NP, a key point on the efficacy of chemotherapeutics, on inhibiting the growth of solid tumors in vivo, is their extent of ‘infiltrating’ established (i.e., large, vascularized) tumors as we have previously reported [16,17]. Large, soft-tissue solid tumors are particularly challenging: cells in deep tumor regions far from vasculature often do not receive sufficient concentrations of therapeutics injected in the blood. We view solid tumors as collectives of avascular regions of different sizes, which we model in vitro by multicellular spheroids. We have previously demonstrated (1) the ability of these spheroids to capture the critical diffusion-limited transport of agents within the tumor interstitium [16,17] and, therefore, (2) that we can use their response to treatments (spheroid size shrinkage and outgrowth inhibition) to predict the relevant efficacy on inhibiting tumors, by same treatments, in vivo [16,17]. To enable this correlation, spheroids were exposed to the various forms of DXR and of CDDR (free and in NP) while also scaling both for the agent concentrations (to be analogous to those concentrations expected in the bloodstream of patients undergoing clinical trials) and for the incubation time of spheroids with each agent form (to be analogous to the times that agents circulating in the blood). We measured the spatiotemporal microdistributions of agents in all forms in spheroids, and we demonstrated that more uniform and greater concentrations of agents (in their bioavailable form, alone and in combination) within spheroids enabled better inhibition of spheroid outgrowth (Figure 5 and Figure 6). Agents delivered by the responsive NP were dramatically more effective in inhibiting spheroid growth than agents delivered by conventional NP (non-responsive NP) and comparable and/or better than agents in their free form. For the latter, the efficacy of the responsive NP refers to comparison to free agents when each form was scaled for its corresponding concentration in the blood and projected circulation time in the blood, as shown in Figure 5C,D of 4.5 vs. 18 μg/mL, for the free forms and the NP forms, respectively; these values were calculated on the basis of both the MTD-constrained blood concentration values of agents, and the corresponding circulation times of the free and the NP forms.

We have previously demonstrated, in mouse models, similar biodistributions for the responsive NP and the non-responsive NP. This was expected given that all NP have similar sizes, zeta potential during circulation in the blood and the same extent of PEGylation [16,17]. Assuming comparable behavior in humans, it would be possible to expect that the responsive NP forms of the two agents may not only exhibit good safety profiles, as the approved NP forms, but may also increase killing efficacy at the tumors.

As with all nanoparticles, clinical applicability will be dependent on the vascular permeability of tumors to NP [33]. In addition, for the responsive NP, intratumoral acidity is necessary—and is common in advanced TNBC [11]—for activating the responsive-NP properties of agent release and/or binding/adhesion to cancer cells or the tumor ECM.

Overall, this study stresses: (1) the significance of the spatiotemporal microdistributions of therapeutics within solid tumors on affecting killing efficacy and (2) the importance of designing drug delivery carriers with properties especially tailored to specific therapeutic agents with the aim to improve the agent’s spatiotemporal microdistributions. While this study focuses on NP engineered for CDDP and for DXR, there are other therapeutics currently being studied in cocktails for TNBC treatment. So long as the NP properties are specifically optimized with the spatiotemporal-delivery needs of their therapeutic cargo in mind, the results shown in this study should hold true for generally improving the therapeutic effect.

## 4. Materials and Methods

### 4.1. Materials

All lipids were purchased from Avanti Polar Lipids (Alabaster, AL, USA), including 1,2-diarachidoyl-sn-glycero-3-phosphocholine (20PC), 1,2-dipalmitoyl-sn-glycero-3-phospho-L-serine (sodium salt) (DPPS), 1,2-distearoyl-sn-glycero-3-phosphoethanolamine-N-[methoxy(polyethylene glycol)-2000] (ammonium salt) (18:0 PEG2000 PE), and 1,2-dipalmitoyl-sn-glycero-3-phosphoethanolamine-N-(lissamine rhodamine B sulfonyl) (ammonium salt) (DPPE-Rhodamine). The adhesive lipid, 1,2-distearoyl-sn-glycero-3-phosphoethanolamine-N-PEG2000-dimenthylammonium propane/propanoyl (DSPE-PEG(2000)-DAP), used in cisplatin-containing, responsive-lipid nanoparticles (responsive-CDDP-NP), was custom synthesized by Avanti Polar lipids [17]. The HER2-targeting lipopeptide (HER2-targeting DPPE-lipopeptide), DPPE-(Gly-Ser-Gly)-Lys-Cys-Cys-Tyr-Ser-Leu, used in doxorubicin-containing lipid nanoparticles (responsive-DXR-NP), was custom synthesized and analyzed by AnaSpec (Fremont, CA, USA) as described before [14]. Doxorubicin hydrochloride (DXR), cis-Diammineplatinum(II) dichloride (CDDP, cisplatin), ammonium sulfate, Poly(2-hydroxyethyl methacrylate) (poly-HEMA), Phosphate Buffered Saline (PBS), Triton-X 100, Sephadex G-50, and Sepharose-4B were purchased from Sigma-Aldrich (St. Louis, MO, USA). Penicillin–Streptomycin, Dulbecco’s Modified Eagle Medium (DMEM), Vybrant^®^ CFDA-SE Cell Tracer Kit (CFDA-SE), and SNARF-4F were purchased from ThermoFisher Scientific (Waltham, MA, USA). Trypsin and Matrigel^®^ Growth Factor Reduced (GFR) Basement Membrane Matrix were purchased from Corning (Corning, NY, USA). Fetal Bovine Serum (FBS) was purchased from Omega Scientific (Tarzana, CA, USA). CellTiter 96^®^ Non-radioactive Cell Proliferation Assay (3-(4,5-Dimethylthiazol-2-yl)-2,5-Diphenyltetrazolium Bromide, MTT). Dye Solution and Solubilization Solution/Stop Mix was purchased from Promega (Madison, WI, USA). 

### 4.2. Cell Lines

The MDA-MB-231 cell line was purchased from the American Type Culture Collection (ATCC, Rockville, MD, USA). The doxorubicin-resistant MDA-MB-231 cell line (DXR-Res-231) was developed as described below. All cells (MDA-MB-231 and DXR-Res-231) were cultured in DMEM, with 10% FBS and 1% Penicillin-Streptomycin added.

### 4.3. Development of Doxorubicin-Resistant MDA-MB-231 Cell Line (DXR-Res-231) 

Almost-confluent flasks of naïve MDA-MB-231 cells were incubated at the measured IC_90_ concentration for free doxorubicin (1.5 µM free DXR, using the MTT assay, vide infra) for three days, then washed thrice with PBS and grown in fresh media. When the surviving cells reached confluency (approximately 3 weeks later), the new IC_90_ of free DXR (3.2 µM) was evaluated, and the process of incubating cells at the new IC_90_ of free DXR was repeated to achieve further chemoresistance [34,35,36]. Finally, on the resulting doxorubicin-resistant MDA-MB-231 (DXR-Res-231) cells, the IC_50_ values of both free DXR and free CDDP were measured. The HER2 expression by both cell lines was measured using the HER2-targeting antibody trastuzumab (as previously reported [14] and also briefly described the Supporting Information, Figure S2). 

### 4.4. Lipid Nanoparticle (NP) Preparation and Characterization

Nanoparticles were formed using the thin-film hydration method as previously described [14,17]. The compositions and properties of responsive and non-responsive DXR-NP and CDDP-NP are shown in Table 5. To form the DXR-NP, DXR was loaded using the ammonium sulfate method. Briefly, lipids were combined in chloroform in a round bottom flask and dried on a rotovap. They were annealed in ammonium sulfate (250 mM, adjusted to pH 7.4) for 2 h, followed by extrusion at a temperature at least 5 °C above the highest transition temperature of all relevant lipids, and finally, were passed through a Sepharose 4B column eluted with PBS. Immediately after eluting from the column, free DXR was added to the NP suspension (at a ratio of 0.5 mM free DXR to 10 micromoles of lipid; 1 mL of 3 mM free DXR (in saline) was added to 1 mL of the NP suspension (15 micromoles lipid/mL PBS)), and the mixture was heated to 80 °C for 2 h. Following completion of loading, NP were allowed to cool to room temperature and then passed through a G50 column to remove unencapsulated free DXR. The loading efficiency was determined by measuring the fluorescence of DXR in NP (DXR excitation/emission = 470/595 nm) before and after separation of non-encapsulated free DXR as follows. NP were diluted in 2 mL of PBS with 100 µL of Triton-X 100 added. Cuvettes were heated at 80 °C until the cloud point of Triton-X 100 was reached, after which they were allowed to cool back to room temperature and measured for fluorescence on a Fluorolog (HORIBA Scientific, Piscataway, NJ, USA). 

To evaluate the HER2-binding property, responsive and non-responsive DXR-NP were incubated with MDA-MB-231 cells (2 million cells/mL) at a ratio of 1:5 DXR-NP–HER2-receptor, at pH 7.4 and 6.5, for six hours in a humidified incubator at 37 °C and 5% CO_2_. After completion of incubation, the fluorescence intensities (Rhodamine excitation/emission = 550/590 nm) associated with cells before and after triple wash with ice-cold PBS, to remove non-cell associated NP, were compared. (Prior to measurement, cells were lysed by a 10 min sonication, after addition of 1mL of acidified Isopropanol in 1mL of cells suspended in water.) 

To form CDDP-NP, CDDP was loaded passively in NP [37]. Briefly, lipids were combined in chloroform, dried, and annealed in PBS for two hours. Following extrusion and purification through a 4B column eluted with PBS, NP (40 μmole lipid in 2 mL PBS) were incubated with CDDP at 17 mg/mL (the solubility limit of CDDP) at 80 °C for four hours (with frequent mixing to resuspend any settled CDDP). Following loading, NP were allowed to cool to room temperature and then centrifuged at 1000 RCF for 10 min to pellet any unencapsulated CDDP. The NP-containing supernatant was removed and passed through a G50 column to separate any remaining unencapsulated CDDP. The loading efficacy was quantified by measuring the Platinum (Pt) absorption at 265.9 nm using an Atomic Absorption Spectrophotometer Graphite Furnace (Buck Scientific Instruments, Norwalk, CT, USA). NP were 4X diluted with 10% HCl, with 100 µL of Triton-X 100 added to release encapsulated CDDP before measuring, and CDDP concentration was determined by comparison to a Pt calibration curve (after verifying lipid content had no effect on Pt signal) [15]. All NP size distributions and zeta potential values were measured using a NanoSeries Zetasizer (Malvern Instruments Ltd., Worcestershire, UK).

To evaluate the release kinetics of doxorubicin from DXR-NP and of cisplatin from CDDP-NP, NP were incubated in DMEM containing 10% FBS at pH 7.4 and pH 6.5 in a humidified incubator at 37 °C and 5% CO_2_. The parent NP suspensions were sampled at different time points, and the released DXR or CDDP was separated by a G50 column. The amount of NP-encapsulated DXR or CDDP was measured as described above, and, finally, a single exponential decay was fit to each release profile. 

### 4.5. Cell Monolayers—Treatment with Single Agents

To evaluate the IC_50_ value for each single agent (i.e., the concentration of a single agent, CDDP or DXR, that inhibited by 50% the survival of cells), in free and/or in NP form, 20,000 cells/well were plated the previous night on a 96-well flat-bottom plate. Cells were incubated with the agent-containing media (DMEM that was preincubated in a humidified incubator at 37 °C and 5% CO_2_ overnight at pH 7.4 or 6.5 to ensure it was fully equilibrated) at different concentrations for six hours (3 wells per concentration at each pH). Following treatment, cells were washed thrice with PBS, then allowed to grow with fresh media for 3 days (corresponding to two doubling times, 36 h). At that point, the MTT assay was used (per manufacturer instructions) to evaluate cell viability. Cell survival vs. agent concentration was fitted using a 3-parameter sigmoid function, and the IC_50_ was determined by calculating the concentration of agent yielding 50% cell viability. 

### 4.6. Cell Monolayers—Treatment with Combination of Agents

DXR and CDDP (in free forms and/or in NP forms) were combined at given mass ratios, and the IC_50_ of the combined agents (in free and/or in NP forms) at each of the mass ratios on cell monolayers was evaluated as described above. At 50% viability of cells (IC_50_ of combination treatment) measured for each ratio of combined agents (in free and/or in NP forms), the concentration of each agent was calculated and was plotted with the CDDP concentration on the x-axis and the DXR concentration on the y-axis. The IC_50_ values of each agent (in free and/or in NP forms) when evaluated as a single treatment (falling directly on each respective axis) were connected by a straight line—this line served to illustrate a purely additive response between the two agents. If a combined agent ratio’s IC_50_ fell above this line, it indicated antagonism between the two agents, while if a combined agent ratio’s IC_50_ fell below this line, it indicated synergism [23,38]. 

### 4.7. Spheroid Formation

All spheroids were formed by centrifuging 250 cells with 2.5% *v/v* Matrigel^TM^ added at 1000 RCF for 10 min on poly-HEMA coated U-bottom 96-well plates. Spheroids were treated when they reached the desired size of 400 µm in diameter, approximately 9 days after seeding. 

### 4.8. Spheroids—Treatment with Single and Combination of Agents

Spheroids were treated with therapeutic agents (DXR and/or CDDP) in free form and/or encapsulated in responsive NP and/or non-responsive NP. Spheroids were incubated with each agent and/or combinations thereof for lengths of time and at concentrations relevant to their blood circulation times and to reported administered doses in clinical studies, respectively. For all forms (free or in NP), the concentration of 18 µg/mL of each therapeutic agent was used—this concentration was extracted from the dose of 50 mg/m^2^ injected I.V. in clinical trials [5,39] that reportedly evaluated combinations of the two chemotherapeutics while scaling by the average human surface area (1.8 m^2^) and blood volume (5 L) [40] (18 µg/mL = 50 mg/m^2^ × 1.8 m^2^/5 L).

The incubation times of spheroids with the different forms of agents were chosen to approximately scale with the corresponding reported residence times in the blood of each agent’s form in humans. In particular, for free CDDP, a one-hour incubation time with spheroids was chosen to mimic the reported infusion kinetics in humans of 1 h, while for free DXR, a 20 min incubation time was used (that equaled the duration of four half-lives of free DXR after the reported single injection of free DXR in humans (NCT02315196). For all NP forms, the incubation time of spheroids was 24 h and was chosen to model the blood clearance half-life of Doxil that has comparable size, zeta potential, and extent of PEGylation to the NP studied herein [26]. 

After treatment, spheroids were transferred into fresh DMEM and allowed to grow. Spheroid volume was tracked until the non-treated spheroids reached a plateau (approximately 14 days later at average size of 700–800 µm diameter), after which spheroids were each plated in individual flat-bottom, adherent plates, where they were allowed to grow until the cells in the non-treated condition reached confluency. At that point, the number of viable cells was counted for each condition and was normalized by the number of cells of the non-treated condition to calculate the percent outgrowth. 

### 4.9. Spatiotemporal Profiles of Agents in Spheroids

For the evaluation of the spatiotemporal profiles of fluorescent surrogates of agents, on MDA-MB-231 spheroids of 400 µm in diameter, CFDA-SE in free form was used as surrogate for the free agents (DXR and CDDP) [17], and CFDA-SE encapsulated in the various forms of NP was used as surrogate of the corresponding NP forms of agents. CFDA-SE was passively loaded into the NP, as previously reported [17]. Spheroids were incubated with NP (2 mM lipid, 0.8 µM CFDA-SE) or with 0.8 µM free CFDA-SE for different duration times depending on the corresponding treatment form (vide supra), and at different time points, spheroids were sampled so as to measure the spatial profiles during the incubation with the fluorophores (uptake) and after transfer of spheroids in fresh media (clearance). At each time point, spheroids were fished, embedded into OCT gel, and frozen at −80 °C. Each spheroid was then sliced into 20 µm thick sections, and the equatorial section was imaged on a Zeiss LSM 780 Confocal Microscope (CFDA excitation/emission = 492/517 nm). The radial spatial profiles of the fluorescent surrogates were generated by analyzing the spheroid fluorescent images using an in-house Matlab erosion code; the averaged intensities of pixels within concentric rings (of 5 µm width) were plotted vs. the spheroid radius for different time points. Finally, the time-integrated concentrations of the fluorescent surrogate over the entire spheroid volume were calculated using the trapezoidal rule. 

### 4.10. Statistical Analysis

All results are reported as the mean ± standard deviation between *n* independent measurements. Significance between treatment conditions was evaluated using the unpaired Student’s *t*-test, with significance defined as *p* < 0.05.

## Figures and Tables

**Table 1:** Characterization of DXR-responsive and non-responsive NP. Values reported as mean ± standard deviation of *n* = 5 independent NP preparations.

DXR-NP (*n* = 5)	Size, nm (PDI)	Zeta Potential (mV)		% Loading Efficiency	Drug-to-Lipid Ratio (*w*/*w*)	% of Cell Associated DXR (6 h Incubation) with MDA-MB-231 Cells		Release Kinetics Fitting Parameters y = y_∞_ + exp(−t/τ _1/2_ )			
								pH 7.4		pH 6.5	
		pH 7.4	pH 6.0			pH 7.4	pH 6.5	y _∞ (%)_	τ_1/2__(min)_	y _∞ (%)_	τ_1/2__(min)_
**Responsive**	162 ± 19 (0.11 ± 0.06)	−5.91 ± 0.60	−5.74 ± 0.89	61 ± 4	0.066 ± 0.011	1.09 ± 0.18	1.71 ± 0.24	90 ± 1.2	66 ± 31	70 ± 1.3	21 ± 4.2
**Non-Responsive**	123 ± 6 (0.09 ± 0.05)	−4.57 ± 0.63	−4.15 ± 0.52	71 ± 8	0.090 ± 0.016	0.79 ± 0.16	0.84 ± 0.31	90 ± 1.3	120 ± 56	90 ± 0.9	54 ± 18

**Table 2:** Characterization for CDDP-responsive and non-responsive NP. Values reported as mean ± standard deviation of *n* = 5 independent NP preparations. ^╪^ for a 50 µmol lipid preparation—passive loading scaled with amount of lipid present.

CDDP-NP (*n* = 5)	Size, nm (PDI)	Zeta Potential (mV)			% Loading Efficiency ^╪^	Drug-to-Lipid Ratio (*w*/*w*)	Release Kinetics Fitting Parameters y = y_∞_ + exp(−t/τ _1/2_ )			
							pH 7.4		pH 6.5	
		pH 7.4	pH 6.5	pH 6.0			y _∞ (%)_	τ_1/2__(min)_	y _∞ (%)_	τ_1/2__(min)_
**Responsive**	123 ± 5 (0.12 ± 0.05)	−2.06 ± 0.41	−0.97 ± 0.46	−0.11± 0.47	5.8 ± 0.98	0.090 ± 0.011	87 ± 1.5	150 ± 45	71 ± 0.5	131 ± 7
**Non-Responsive**	115 ± 6 (0.10 ± 0.04)	−4.57 ± 0.63	−4.48 ± 0.56	−4.15 ± 0.52	6.2 ± 0.73	0.103 ± 0.054	89 ± 0.3	133 ± 10	88 ± 0.3	157 ± 14

**Table 3:** Characterization of MDA-MB-231 (ATCC) and the doxorubicin-resistant MDA-MB-231, DXR-Res-231, cell lines. Doubling time and IC_50_ of free agents reported as mean ± standard deviation between *n* = 3 independent measurements. HER2 expression and K_D_ reported as the fitting parameter ± error, as shown in Supplemental Figure S2. The degree of resistance is defined as the IC_50_ of free agents on resistant cells divided by the IC_50_ on naïve MDA-MB-231 (ATCC) cells.

Cell Line Characterization	Doubling Time (h)	HER-2 Expression, Receptors per Cell (K_D_, nM)	IC_50_ of Free DXR (µg/mL)		IC_50_ of Free CDDP (µg/mL)	
			pH 7.4	pH 6.5	pH 7.4	pH 6.5
**MDA-MB-231** **(ATCC)**	>36 ± 3	>83,345 ± 10,117 (8.45 ± 3.81)	>1.20 ± 0.14	>3.74 ± 0.31	>8.82 ± 1.66	>9.73 ± 2.63
**DXR-Res-231**	34 ± 4	77,202 ± 7166 (8.00 ± 2.80)	2.57 ± 0.90	9.52 ± 2.95	9.67 ± 2.07	9.63 ± 0.61
**Degree of Resistance** **(IC_50_ Resistant/IC_50_ Naïve)**			2.1 ± 0.8	2.5 ± 0.8	1.1 ± 0.3	1.0 ± 0.3

**Table 4:** IC_50_ values of responsive and non-responsive DXR-NP and of CDDP-NP on MDA-MB-231 and DXR-Res-231 cells. Values reported as mean ± standard deviation of *n* = 3 independent NP preparations. ^╪^ indicates 50% kill of cell population was not reached (up to the maximum incubation concentration of 680 µg/mL of DXR encapsulated in non-responsive DXR-NP).

	**IC_50_ of Responsive-DXR-NP** **(µg/mL)**		**IC_50_ of Responsive-CDDP-NP** **(µg/mL)**		**IC_50_ of Non-Responsive-DXR-NP** **(µg/mL)**		**IC_50_ of Non-Responsive-CDDP-NP** **(µg/mL)**	
	**pH 7.4**	**pH 6.5**	**pH 7.4**	**pH 6.5**	**pH 7.4**	**pH 6.5**	**pH 7.4**	**pH 6.5**
**MDA-MB-231** **(ATCC)**	62 ± 21	35 ± 11	155 ± 101	63 ± 62	195 ± 67	575 ± 290	716 ± 25	721 ± 8
**DXR-Res-231**	75 ± 30	37 ± 20	154 ± 15	61 ± 7	Not measurable ^╪^		713 ± 52	761 ± 48

**Table 5:** NP compositions and properties. The ‘+’ sign indicates that NP exhibit the listed property, while the ‘−’ sign indicates that the property is absent. Composition is reported as mole percent.

NP Compositions (Mole %)		pH- Triggered Content Release	ECM Adhesion	HER2 Targeting	20PC	DPPS	Cholesterol	DSPE-18PEG	DPPE-Rhodamine	PEG-DAP	HER2-Targeting Lipopeptide
**DXR-** **NP**	**Responsive**	**+**	**−**	**+**	81	9	4.5	4	0.5	−	1
	**Non-Responsive**	**−**	**−**	**−**	76.5	−	19	4	0.5	−	−
**CDDP-NP**	**Responsive**	**+**	**+**	**−**	53	35	4.5	−	0.5	7	−
	**Non-Responsive**	**−**	**−**	**−**	73.5	−	19	7	0.5	−	−

## Data Availability

The data presented in this study are available in the Supplementary Materials.

## Supplementary Materials

The following are available online at https://www.mdpi.com/article/10.3390/ph14101035/s1, Table S1: Viability of non-treated cells from IC_50_ experiments at extracellular pH values of 7.4 and 6.5, Table S2: IC_50_ values of free DXR and free CDDP given in combination on MDA-MB-231 and (the ‘doxorubicin resistant’) DXR-Res-231 cell lines, Table S3: Combination index (CI) of free DXR and free CDDP on MDA-MB-231 and DXR-Res-231 cell lines at extracellular pH 7.4 and 6.5, Table S4: IC_50_ values of DXR and of CDDP delivered, in combination, by responsive-NP on MDA-MB-231 and DXR-Res-231 cell lines, Table S5: Combination index (CI) of DXR and of CDDP delivered, in combination, by responsive-NP on MDA-MB-231 and DXR-Res-231 cell lines at extracellular pH 7.4 and 6.5, Table S6: IC_50_ values and combination index values of DXR and of CDDP delivered, in combination, by non-responsive-NP on MDA-MB-231 cell line, Figure S1: Release kinetics of responsive- and non-responsive DXR- and CDDP- NP at pH 7.4 and 6.5, Figure S2: K_D_ of trastuzumab binding to the HER2 receptors on MDA-MB-231 and DXR-Res-231 cell lines, Figure S3: Individual free agent IC_50_ values of DXR and of CDDP on MDA-MB-231 and DXR-Res-231 at pH 7.4 and 6.5, Figure S4: Flow Cytometry Demonstrating Relative HER2-Receptor Expression by Cells, Figure S5: IC_50_ plots of responsive and non-responsive NP on the MDA-MB-231 (ATCC) cell line, Figure S6: IC_50_ plots of responsive (black symbols) and non-responsive (white symbols) NP on the (‘doxorubicin-resistant’) DXR-Res-231 cell line, Figure S7: IC_50_ measurements of responsive-NP not containing any of the chemotherapeutic agents, Figure S8: Normalized IC_50_ values of free DXR and free CDDP introduced alone and in combination on MDA-MB-231 and DXR-Res-231 cell lines, Figure S9: Normalized IC_50_ values of DXR and of CDDP delivered, in combination, by responsive-NP on MDA-MB-231 and DXR-Res-231 cell lines, Figure S10: Normalized IC_50_ values of DXR and of CDDP delivered, in combination, by responsive- and non-responsive-NP on the MDA-MB-231 cell line, Figure S11: Measured extracellular pH of spheroids formed by MDA-MB-231 and DXR-Res-231, Figure S12: Spatiotemporal microdistributions of the fluorescent drug surrogate CFDA-SE on MDA-MB-231 spheroids delivered by responsive-NP, designed for CDDP and for DXR, and of non-responsive NP, Figure S13: Spatiotemporal microdistributions of the free fluorophore CFDA-SE, used as surrogate of the free chemotherapeutic agents, on MDA-MB-231 spheroids.
