# Supplementary material for: Growth Inhibition of Triple-Negative Breast Cancer: The Role of Spatiotemporal Delivery of Neoadjuvant Doxorubicin and Cisplatin
Source: Pharmaceuticals (Basel). 2021 Oct 12;14(10):1035. doi: 10.3390/ph14101035 (PMC8540483; doi:10.3390/ph14101035)
Supplement: Supplementary file 1 [file pharmaceuticals-14-01035-s001.zip › pharmaceuticals-1381365-supplementary.pdf]

SUPPLEMENTAL INFORMATION

n=6	MTT Measured Absorbance Value		% Cell viability relative to pH 7.4	
	**pH 7.4**	**pH 6.5**	**pH 7.4**	**pH 6.5**
MDA-MB-231	0.54 ± 0.02	0.50 ± 0.06	100 ± 4%	**93 ± 7%**
DXR-Res-231	0.55 ± 0.03	0.52 ± 0.05	100 ± 5%	**95 ± 7%**

Table S1. Viability of non-treated cells from IC_{50} experiments at extracellular pH values of 7.4 and 6.5. A statistically significant decrease in the viability of untreated cells was not observed as a result of the decrease in extracellular pH during the 6 hour incubation during these studies.

Free Agents

n = 3	MDA-MB-231 (ATCC)				DXR-Res-231			
	IC50 of Free CDDP (µg/mL)		IC50 of Free DXR (µg/mL)		IC50 of Free CDDP (µg/mL)		IC50 of Free DXR (µg/mL)	
Mass Ratio CDDP:DXR	pH 7.4	pH 6.5	pH 7.4	pH 6.5	pH 7.4	pH 6.5	pH 7.4	pH 6.5
0 : 1	N/A	N/A	1.20 ± 0.14	3.74 ± 0.31	N/A	N/A	2.57 ± 0.90	9.52 ± 2.95
1 : 2	0.72 ± 0.44	1.53 ± 0.65	1.09 ± 0.66	2.30 ± 0.98	0.82 ± 0.18	1.60 ± 0.20	1.63 ± 0.35	3.20 ± 0.40
1 : 1	0.59 ± 0.18	1.55 ± 0.43	0.59 ± 0.18	1.55 ± 0.43	1.20 ± 0.10	2.33 ± 0.31	1.20 ± 0.10	2.33 ± 0.31
2 : 1	1.4 ± 0.48	2.85 ± 0.14	0.47 ± 0.16	0.95 ± 0.05	2.27 ± 0.64	4.46 ± 1.10	1.13 ± 0.32	2.23 ± 0.55
5 : 1	2.48 ± 1.08	4.29 ± 1.32	0.23 ± 0.10	0.39 ± 0.12	4.80 ± 1.13	6.43 ± 2.08	0.96 ± 0.23	1.29 ± 0.42
10 : 1	5.06 ± 1.29	5.83 ± 2.46	0.46 ± 0.12	0.53 ± 0.22	6.67 ± 3.04	8.53 ± 3.40	0.67 ± 0.30	0.85 ± 0.34
20 : 1	4.42 ± 1.29	6.54 ± 2.88	0.21 ± 0.06	0.31 ± 0.14	7.36 ± 2.08	9.10 ± 1.82	0.39 ± 0.10	0.46 ± 0.09
1 : 0	8.83 ± 1.66	9.73 ± 2.63	N/A	N/A	9.67 ± 2.07	9.63 ± 0.61	N/A	N/A

Table S2. IC_{50} values of **free DXR and free CDDP** given in combination on MDA-MB-231 and (the 'doxorubicin resistant') DXR-Res-231 cell lines across a range of different CDDP:DXR mass ratios following a 6 hour incubation. Values reported as mean ± standard deviation between n=3 independent measurements (with n=3 samples per condition per measurement).

Free Agents

n = 3	MDA-MB-231 (ATCC)		DXR-Res-231	
Mass Ratio CDDP:DXR	pH 7.4	pH 6.5	pH 7.4	pH 6.5
0 : 1	1.0 ± 0.12	1.0 ± 0.08	1.0 ± 0.35	1.0 ± 0.31
1 : 2	0.98 ± 0.30	0.99 ± 0.33	0.71 ± 0.15	0.50 ± 0.06
1 : 1	0.55 ± 0.16	0.56 ± 0.16	0.59 ± 0.05	0.49 ± 0.06
2 : 1	0.54 ± 0.19	0.55 ± 0.03	0.67 ± 0.19	0.69 ± 0.17
5 : 1	0.47 ± 0.20	0.55 ± 0.17	0.87 ± 0.20	0.80 ± 0.26
10 : 1	0.95 ± 0.24	0.74 ± 0.31	0.95 ± 0.43	0.98 ± 0.39
20 : 1	0.68 ± 0.19	0.76 ± 0.33	0.90 ± 0.25	0.99 ± 0.19
1 : 0	1.0 ± 0.19	1.0 ± 0.27	1.0 ± 0.21	1.0 ± 0.06

Table S3. Combination index (CI) of **free DXR and free CDDP** on MDA-MB-231 and DXR-Res-231 cell lines at extracellular pH 7.4 and 6.5. The CI is defined as the sum of the normalized IC_{50} values at each ratio (CI = [(IC_{50} of free CDDP in combination treatment) / (IC_{50} of free CDDP alone)] + [(IC_{50} of free DXR in combination treatment) / (IC_{50} of free DXR alone)]). If the CI is greater than 1, it indicates antagonism between the two agents, while if the CI is less than 1, it indicates synergism. Values are reported as the mean ± standard deviation between n=3 independent measurements.

Responsive-NP

	MDA-MB-231 (ATCC)				DXR-Res-231			
n = 3	IC50 of CDDP-NP (µg/mL)		IC50 of DXR-NP (µg/mL)		IC50 of CDDP-NP (µg/mL)		IC50 of DXR-NP (µg/mL)	
Mass Ratio Encapsulated CDDP:DXR	pH 7.4	pH 6.5	pH 7.4	pH 6.5	pH 7.4	pH 6.5	pH 7.4	pH 6.5
**0 : 1**	N/A	N/A	62 ± 21	35 ± 11	N/A	N/A	75 ± 30	37 ± 20
**1 : 2**	13 ± 5	22 ± 5	27 ± 11	43 ± 11	21 ± 4	20 ± 10	42 ± 8	40 ± 20
**1 : 1**	18 ± 3	27 ± 5	18 ± 3	27 ± 5	32 ± 8	28 ± 8	32 ± 9	28 ± 8
**2 : 1**	26 ± 6	29 ± 8	9 ± 2	10 ± 3	47 ± 15	28 ± 2	16 ± 5	9 ± 1
**5 : 1**	64 ± 8	62 ± 25	11 ± 2	10 ± 4	74 ± 11	43 ± 4	12 ± 2	7 ± 1
**10 : 1**	66 ± 18	49 ± 3	6 ± 2	4 ± 1	93 ± 10	50 ± 8	8 ± 1	5 ± 1
**20 : 1**	127 ± 48	71 ± 19	6 ± 2	3 ± 1	111 ± 9.9	55 ± 9	5 ± 0.5	3 ± 0.5
**1 : 0**	155 ± 101	63 ± 62	N/A	N/A	154 ± 15	61 ± 7	N/A	N/A

Table S4. IC_{50} values of DXR and of CDDP delivered, in combination, by **responsive-NP** on MDA-MB-231 and DXR-Res-231 cell lines across a range of different CDDP:DXR mass ratios following a 6 hour incubation. Values reported as mean ± standard deviation between n=3 independent measurements (with n=3 samples per condition per measurement).

Responsive-NP

n = 3	MDA-MB-231 (ATCC)		DXR-Res-231	
Mass Ratio Encapsulated CDDP:DXR	pH 7.4	pH 6.5	pH 7.4	pH 6.5
0 : 1	1.0 ± 0.35	1.0 ± 0.32	1.0 ± 0.39	1.0 ± 0.54
1 : 2	0.51 ± 0.20	1.54 ± 0.39	0.70 ± 0.14	1.41 ± 0.70
1 : 1	0.41 ± 0.06	1.2 ± 0.24	0.64 ± 0.17	1.19 ± 0.34
2 : 1	0.30 ± 0.07	0.74 ± 0.21	0.51 ± 0.17	0.70 ± 0.05
5 : 1	0.58 ± 0.07	1.28 ± 0.52	0.65 ± 0.10	0.90 ± 0.09
10 : 1	0.52 ± 0.15	0.90 ± 0.06	0.72 ± 0.08	0.95 ± 0.16
20 : 1	0.91 ± 0.35	1.22 ± 0.34	0.79 ± 0.07	0.98 ± 0.15
1 : 0	1.0 ± 0.66	1.0 ± 0.98	1.0 ± 0.09	1.0 ± 0.12

Table S5. Combination index (CI) of DXR and of CDDP delivered, in combination, by **responsive-NP** on MDA-MB-231 and DXR-Res-231 cell lines at extracellular pH 7.4 and 6.5. The CI is defined as the sum of the normalized IC_{50} values at each ratio (CI = [(IC_{50} of free CDDP in combination treatment) / (IC_{50} of free CDDP alone)] + [(IC_{50} of free DXR in combination treatment) / (IC_{50} of free DXR alone)]). If the CI is greater than 1, it indicates antagonism between the two agents, while if the CI is less than 1, it indicates synergism. Values are reported as the mean ± standard deviation between n=3 independent measurements.

Non-Responsive-NP

	MDA-MB-231 (ATCC)					
n = 2	IC50 of CDDP-NP (µg/mL)		IC50 of DXR-NP (µg/mL)		Combination Index	
Mass Ratio Encapsulated CDDP:DXR	pH 7.4	pH 6.5	pH 7.4	pH 6.5	pH 7.4	pH 6.5
0 : 1	N/A	N/A	195 ± 67	575 ± 290	1.0 ± 0.49	1.0 ± 0.72
1 : 2	119 ± 36	183 ± 57	237 ± 72	366 ± 113	1.38 ± 0.42	0.89 ± 0.28
1 : 1	223 ± 23	366 ± 90	223 ± 23	366 ± 90	1.45 ± 0.15	1.15 ± 0.28
2 : 1	341 ± 38	462 ± 46	114 ± 13	155 ± 15	1.06 ± 0.12	0.91 ± 0.09
5 : 1	430 ± 3	553 ± 25	72 ± 1	92 ± 4	0.97 ± 0.01	0.93 ± 0.02
10 : 1	454 ± 49	622 ± 11	41 ± 4	57 ± 1	0.85 ± 0.10	0.96 ± 0.02
20 : 1	611 ± 23	731 ± 73	29 ± 2	35 ± 3	1.0 ± 0.04	1.08 ± 0.10
1 : 0	716 ± 25	721 ± 8	N/A	N/A	1.0 ± 0.03	1.0 ± 0.02

Table S6. IC_{50} values and combination index values of DXR and of CDDP delivered, in combination, by **non-responsive-NP** on MDA-MB-231 cell line across a range of different CDDP:DXR mass ratios following a 6 hour incubation. Values reported as mean ± standard deviation between n=2 independent measurements (with n=3 samples per condition per measurement). The CI is defined as the sum of the normalized IC_{50} values at each ratio (CI = [(IC_{50} of free CDDP in combination treatment) / (IC_{50} of free CDDP alone)] + [(IC_{50} of free DXR in combination treatment) / (IC_{50} of free DXR alone)]). If the CI is greater than 1, it indicates antagonism between the two agents, while if the CI is less than 1, it indicates synergism. Values are reported as the mean ± standard deviation between n=3 independent measurements.

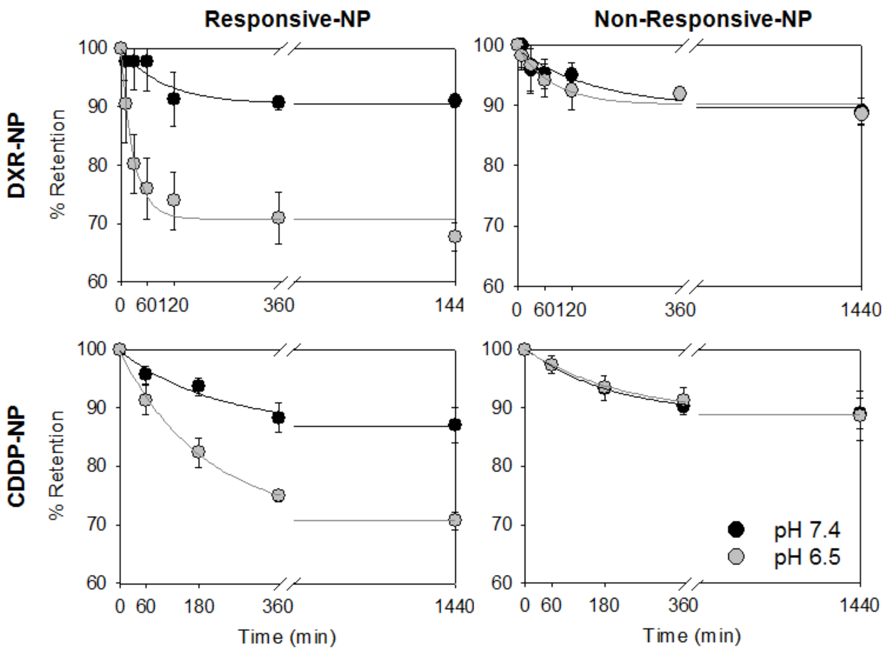

$$y = y_{\infty} + \exp(-t / \tau_{1/2})$$

DXR-NP (n=3)	pH	y_{∞}(%)	$\tau_{1/2}$(min)
Responsive	7.4	90 ± 1.2	66 ± 31
	6.5	70 ± 1.3	21 ± 4.2
Non-Responsive	7.4	90 ± 1.3	120 ± 56
	6.5	90 ± 0.9	54 ± 18

CDDP-NP (n=3)	pH	y_{∞}(%)	$\tau_{1/2}$(min)
Responsive	7.4	87 ± 1.5	150 ± 45
	6.5	71 ± 0.5	131 ± 7
Non-Responsive	7.4	89 ± 0.3	133 ± 10
	6.5	88 ± 0.3	157 ± 14

Figure S1. Release kinetics of responsive- and non-responsive DXR- and CDDP- NP at pH 7.4 (black symbols) and 6.5 (grey symbols). Error bars correspond to the standard deviation between n=3 measurements. Tables list the parameters (and corresponding errors) from fitting a single exponential decay equation to each set of data.

Method

To evaluate the release kinetics of DXR- and CDDP-NP, NP were incubated in DMEM containing 10% FBS at pH 7.4 and pH 6.5 in a humidified incubator at 37°C and 5% CO_2. An aliquot was removed at each time point and passed through a G50 column. The NP were collected, and the retained encapsulated DXR or CDDP was measured. Finally, a single exponential decay was fit to each condition.

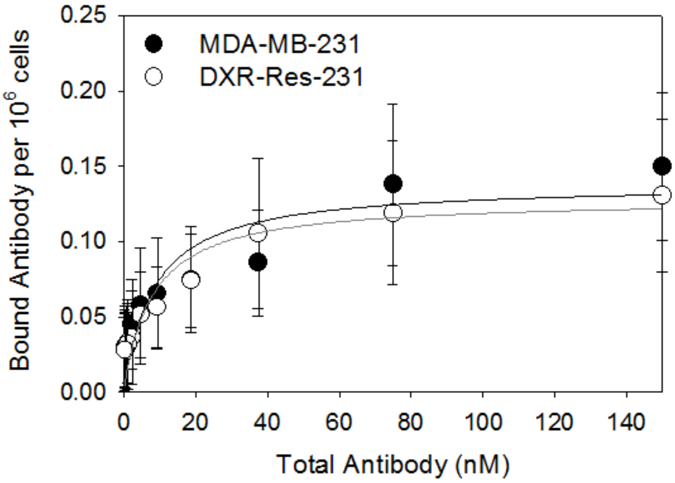

Figure S2. K_D of trastuzumab binding to the HER2 receptors on MDA-MB-231(black symbols) and DXR-Res-231 (grey symbols) cell lines. Error bars correspond to standard deviation between n=2 samples per condition.

Method

MDA-MB-231 and DXR-Res-231 cells were allowed to rotate in tubes on ice at a concentration of 1 million cells/mL. To each tube, varying concentrations of Indium-111 radiolabeled, HER2-targeting trastuzumab (specific activity: 1 µCi/µg, immunoreactivity: 76%) were added, and allowed to incubate on ice for one hour until equilibrium was reached. A second set of tubes, pre-incubated with 50 times excess non-radiolabeled trastuzumab, was treated in parallel with radiolabeled trastuzumab to account for non-specific binding. After completion of incubation, all tubes were washed thrice with ice cold PBS, and the remaining cell-associated radioactivity was measured on a Gamma-counter (Packard Cobra II Auto-Gamma, Model E5003, 50 -550eV range). The non-specific binding values were subtracted from their corresponding total unblocked binding values, and a single rectangular hyperbola was fit to the resulting specific binding curve.

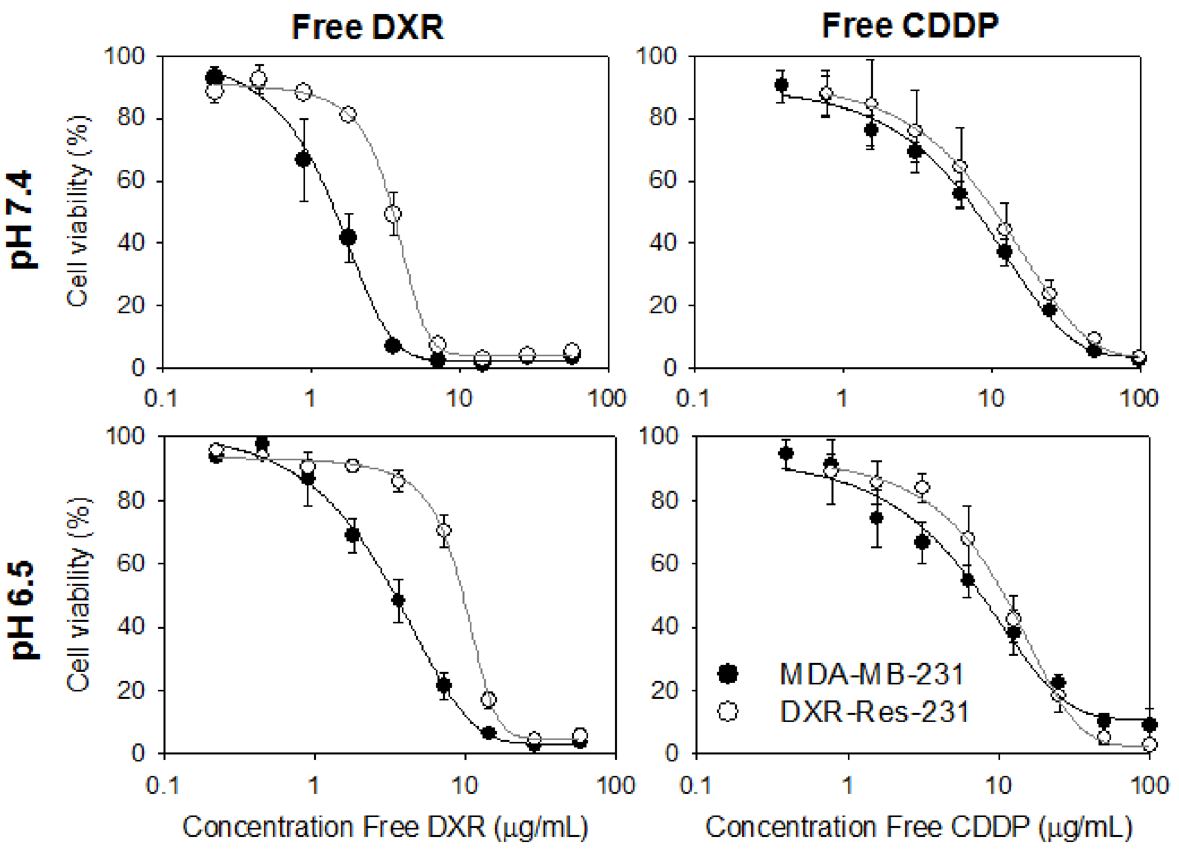

Figure S3. Individual free agent IC_{50} values of DXR and of CDDP on MDA-MB-231 (black symbols) and DXR-Res-231 (white symbols) at pH 7.4 and 6.5. Plotted points and error bars correspond to the average and standard deviation between n=3 independent measurements (with n=3 samples per condition per measurement). These plots serve to illustrate (1) for each cell line independently, the efficacy of free DXR decreased at lower pH while free CDDP efficacy remained unaffected by pH, and (2) the overall decreased efficacy of free DXR on the DXR-Res-231 cell line when compared to the naïve MDA-MB-231 cell line, with free CDDP efficacy remaining the same.

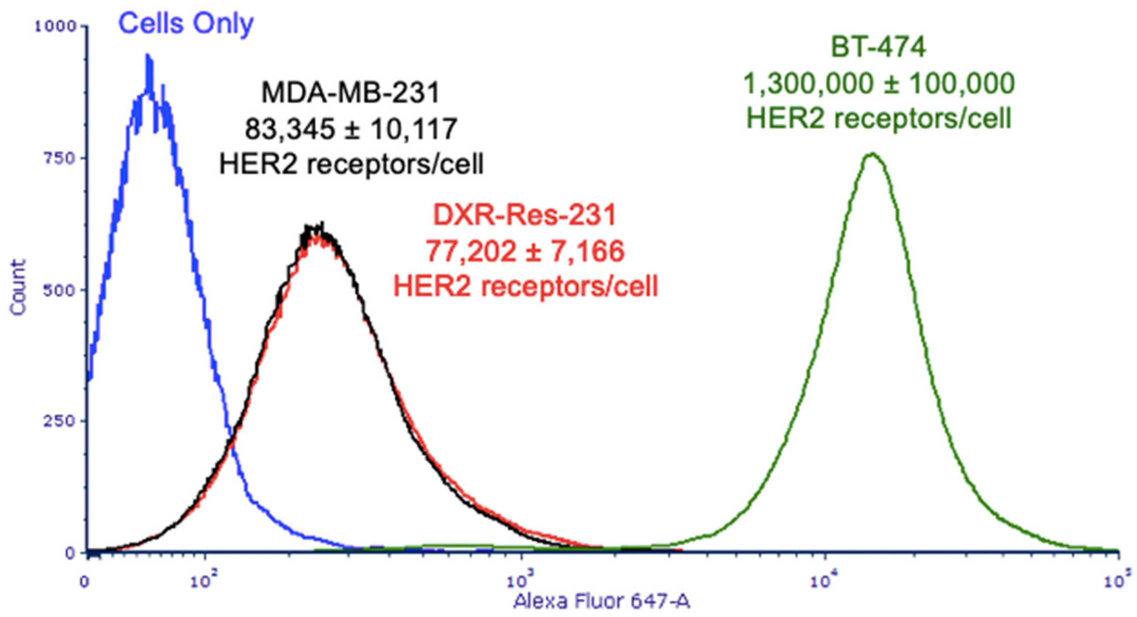

Figure S4. Flow Cytometry Demonstrating Relative HER2-Receptor Expression by Cells. MDA-MB-231 (black line), DXR-Res-231 (red line), and BT-474 (green line; with known high levels of HER2 expression) were incubated on ice for one hour with Alexa-Fluor-647 labeled Trastuzumab (immunoreactivity = 71%). The "cells only" condition (blue line) was not incubated with antibody, to account for autofluorescence from cells. Following washing, cells were resuspended at 1 million cells/mL and run on a BD FACSCanto (Becton Dickinson, Franklin Lakes, New Jersey) to measure Alexa-Fluor-647 fluorescence (ex/em: 650/670 nm). After gating to account for single cells only, fluorescence distributions were plotted, and peak values of each cell line correlated with average receptor expression values per cell that were measured from the binding curve (K_D) experiments (for MDA-MB-231 and DXR-Res-231, please see Table 3 and Figure S2; for high HER2-expressing BT-474 cells please see reference [1]).

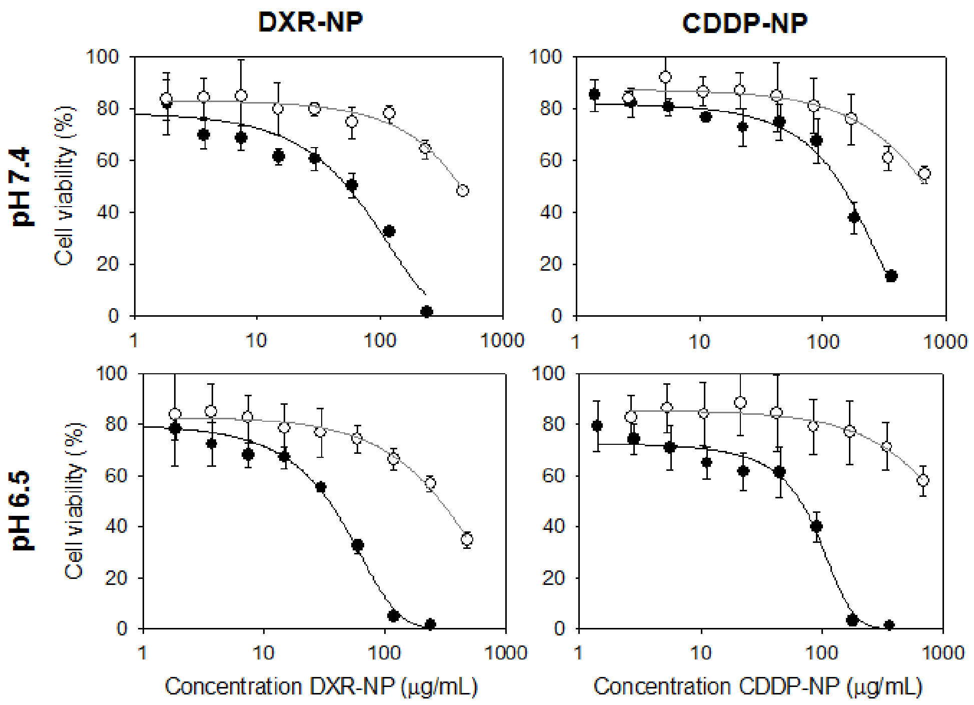

MDA-MB-231 (ATCC)	IC50 of DXR-NP (µg/mL)		IC50 of CDDP-NP (µg/mL)	
	pH 7.4	pH 6.5	pH 7.4	pH 6.5
**Responsive (n=3)**	62 ± 21	35 ± 11	155 ± 101	63 ± 62
**Non-Responsive (n=2)**	195 ± 67	575 ± 290	716 ± 25	721 ± 8

● responsive-NP
○ non-responsive-NP

Figure S5. IC_{50} plots of responsive (black symbols) and non-responsive (white symbols) NP on the **MDA-MB-231** (ATCC) cell line. Plotted points and error bars correspond to the average and standard deviation between n=3 or n=2 independent measurements (with n=3 samples per condition per measurement), with resulting IC_{50} values presented in the table below. These plots serve to illustrate (1) under all conditions, responsive-NP were significantly more effective than non-responsive NP, and (2) responsive-NP increased their efficacy at lower pH, due to the increase in bioavailable therapeutic agent resulting from the increase in NP content release.

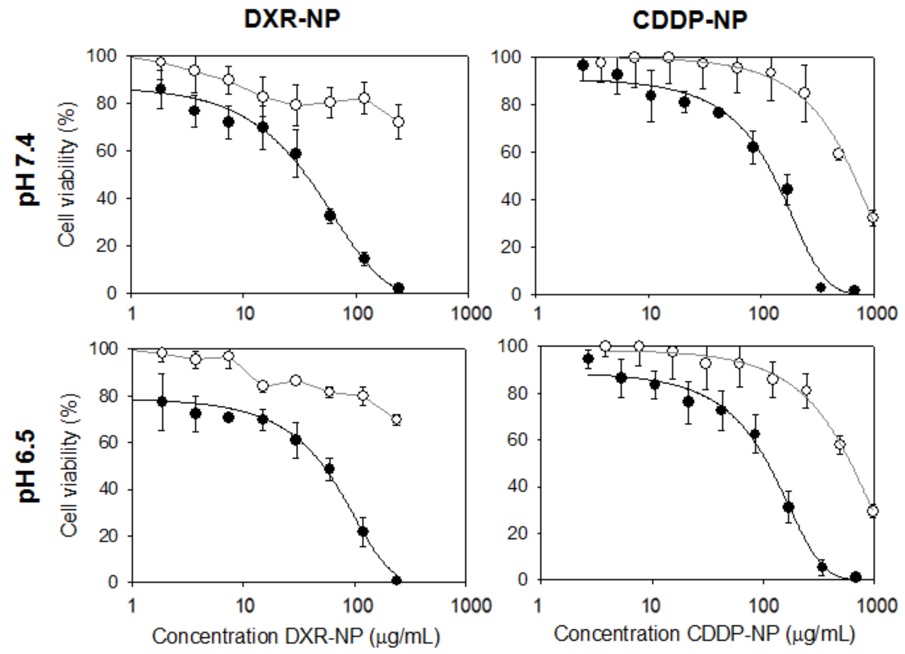

DXR-Res-231	IC50 of DXR-NP (µg/mL)		IC50 of CDDP-NP (µg/mL)	
	pH 7.4	pH 6.5	pH 7.4	pH 6.5
Responsive (n=3)	75 ± 30	37 ± 20	154 ± 15	61 ± 7
Non-Responsive (n=2)	not measurable		713 ± 52	761 ± 48

Figure S6. IC_{50} plots of responsive (black symbols) and non-responsive (white symbols) NP on the ('doxorubicin-resistant') **DXR-Res-231** cell line. Plotted points and error bars correspond to the average and standard deviation between n=3 or n=2 independent measurements (with n=3 samples per condition per measurement), with resulting IC_{50} values presented in the table below. For non-responsive-DXR-NP, values listed as "not measurable" indicate 50% killing of cell population was not reached (up to maximum incubation concentration of 680µg/mL non-responsive-DXR-NP). These plots serve to illustrate (1) under all conditions, responsive-NP were significantly more effective than non-responsive NP, and (2) responsive-NP increased their efficacy at lower pH, due to the increase in bioavailable therapeutic agent resulting from the increase in NP content release.

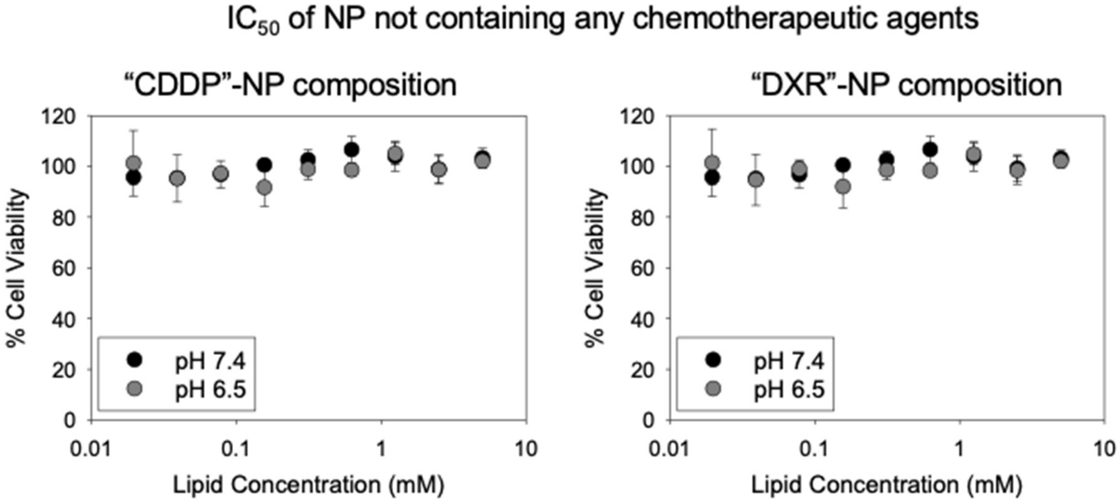

Figure S7. IC_{50} measurements of responsive-NP not containing any of the chemotherapeutic agents. Maximum incubation concentration of 5mM lipid corresponds to the maximum concentration of lipid that was used in responsive-(drug-loaded)-NP IC_{50} measurements shown in Figure 3. All measured cell viabilities were within error of the non-treated cells (n=3 wells per lipid concentration).

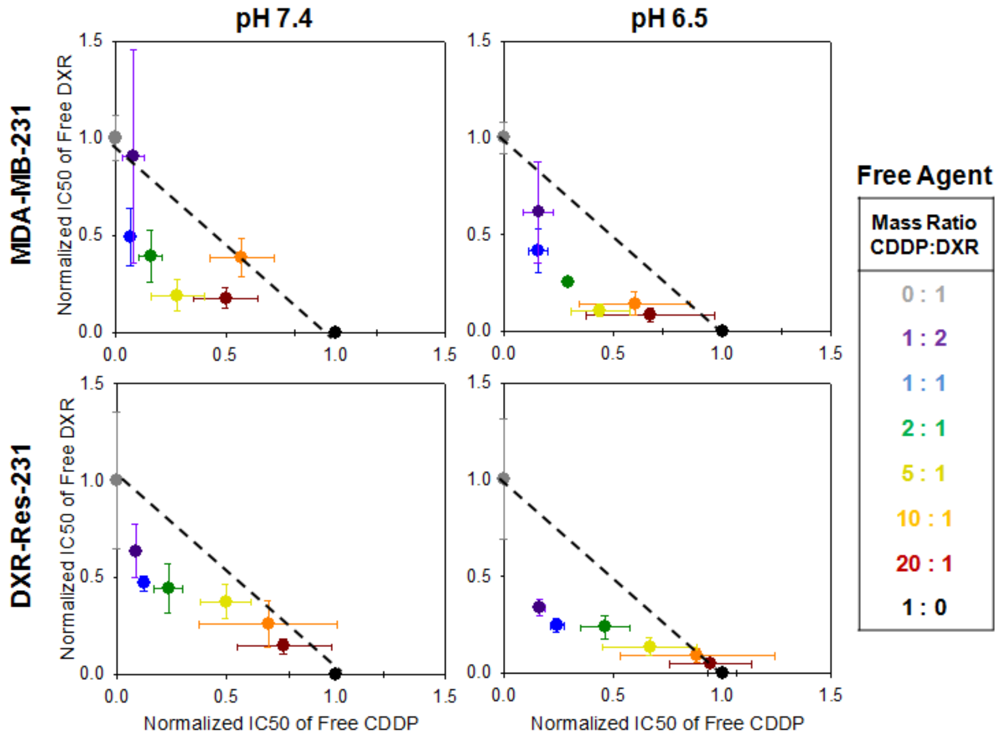

Figure S8. MDA-MB-231 and DXR-Res-231 cell lines. Normalized IC_{50} values of **free DXR and free CDDP** introduced alone and in combination on MDA-MB-231 and DXR-Res-231 cell lines at pH 7.4 (left column) and pH 6.5 (right column) across a range of different CDDP:DXR mass ratios following a 6 hour incubation. To better quantify the synergism/antagonism, the combination IC_{50} plots (Figure 2 in Main Text) were normalized by the respective single agent IC_{50} value, adjusting the "additive effect" line to intersect each axis at 1 by the equation: [(IC_{50} of CDDP in ratio) / (IC_{50} of CDDP alone) for the x-axis, and (IC_{50} of DXR in ratio) / (IC_{50} of DXR alone) for the y-axis]. The dotted lines serve as guide to the eye connecting the single agent normalized IC_{50} values (defined as 1.0 on each axis) and illustrate a purely additive relationship between the two agents. If a point falls above this line, it indicates antagonism between the two agents, while if a point falls below this line it indicates synergism. Error bars correspond to standard deviation of n=3 independent measurements.

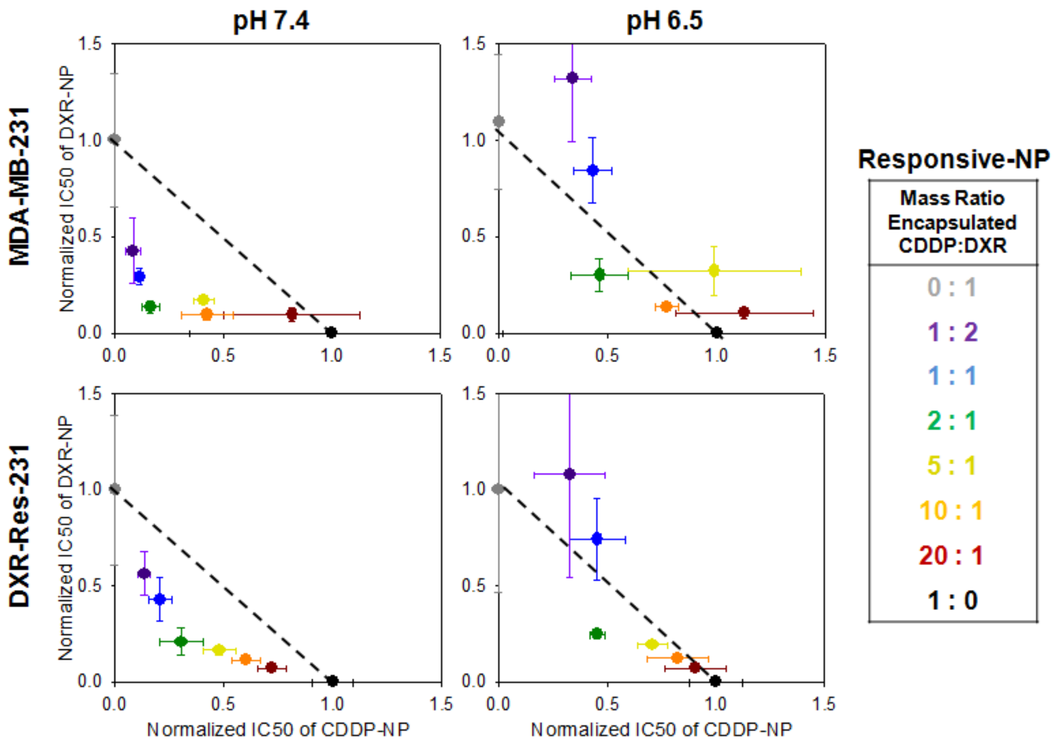

Figure S9. MDA-MB-231 and DXR-Res-231 cell lines. Normalized IC_{50} values of DXR and of CDDP delivered, in combination, by **responsive-NP** on MDA-MB-231 and DXR-Res-231 cell lines across a range of different encapsulated CDDP:DXR mass ratios following a 6 hour incubation. To better quantify the synergism/antagonism, the combination IC_{50} plots (Figure 3 in Main Text) were normalized by the respective single agent IC_{50} value, adjusting the "additive effect" line to intersect each axis at 1 by the equation: [(IC_{50} of CDDP in ratio) / (IC_{50} of CDDP alone) for the x-axis, and (IC_{50} of DXR in ratio) / (IC_{50} of DXR alone) for the y-axis]. The dotted lines serve as guide to the eye connecting the single agent normalized IC_{50} values (defined as 1.0 on each axis) and illustrate a purely additive relationship between the two agents. If a point falls above this line, it indicates antagonism between the two agents, while if a point falls below this line it indicates synergism. Error bars correspond to standard deviation of n=3 independent measurements.

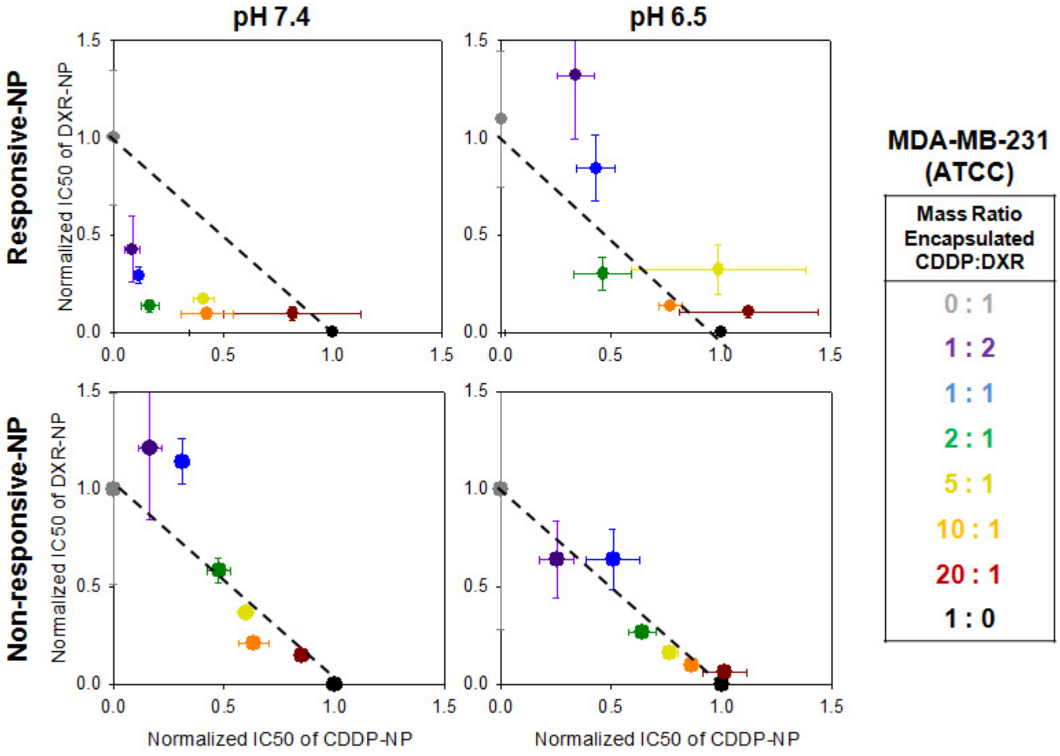

Figure S10. MDA-MB-231 cell line. Normalized IC_{50} values of DXR and of CDDP delivered, in combination, by **responsive- and non-responsive-NP** on the MDA-MB-231 cell line across a range of different encapsulated CDDP:DXR mass ratios following a 6 hour incubation. To better quantify the synergism/antagonism, the combination IC_{50} plots (Figure 4 in Main Text) were normalized by the respective single agent IC_{50} value, adjusting the "additive effect" line to intersect each axis at 1 by the equation: [(IC_{50} of CDDP in ratio) / (IC_{50} of CDDP alone) for the x-axis, and (IC_{50} of DXR in ratio) / (IC_{50} of DXR alone) for the y-axis]. The dotted lines serve as guide to the eye connecting the single agent normalized IC_{50} values (defined as 1.0 on each axis) and illustrate a purely additive relationship between the two agents. If a point falls above this line, it indicates antagonism between the two agents, while if a point falls below this line it indicates synergism. Error bars correspond to standard deviation of n=3 independent measurements.

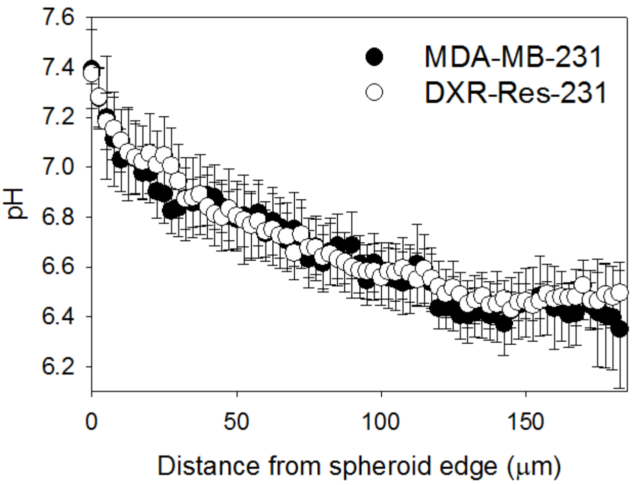

Figure S11. Measured extracellular pH of spheroids formed by MDA-MB-231 (closed symbols) and DXR-Res-231 (open symbols). Error bars correspond to the standard deviation of independent measurements on n=3 different spheroids.

Method (adapted from Reference [2])

Spheroids (380μm diameter) were incubated with the membrane impermeant pH indicator SNARF-4F (350 μM) in media overnight in a humidified incubator at 37°C and 5% CO_2. The ratio of SNARF-4F's emission intensities at 640 nm and 580 nm (ex: 514 nm) varies linearly with pH between the values 6 to 8 independent of the fluorophore's concentration. Immediately before measurement, spheroids were placed in fresh media and imaged on a Zeiss LSM 780 confocal microscope (10X objective, optical slices of z=10 μm). For simplicity, only radial dependence was assumed. An in-house developed Matlab erosion algorithm (with concentric ring width equal to 2.5μm) was used on the images of the equatorial optical slices to evaluate the mean radial intensities and to generate the ratios of the intensities as a function of position. A calibration curve of SNARF-4F in media at known pH values (in a flat cuvette of 10μm path length, imaged using the same microscope) was used to convert the intensity ratios to pH values.

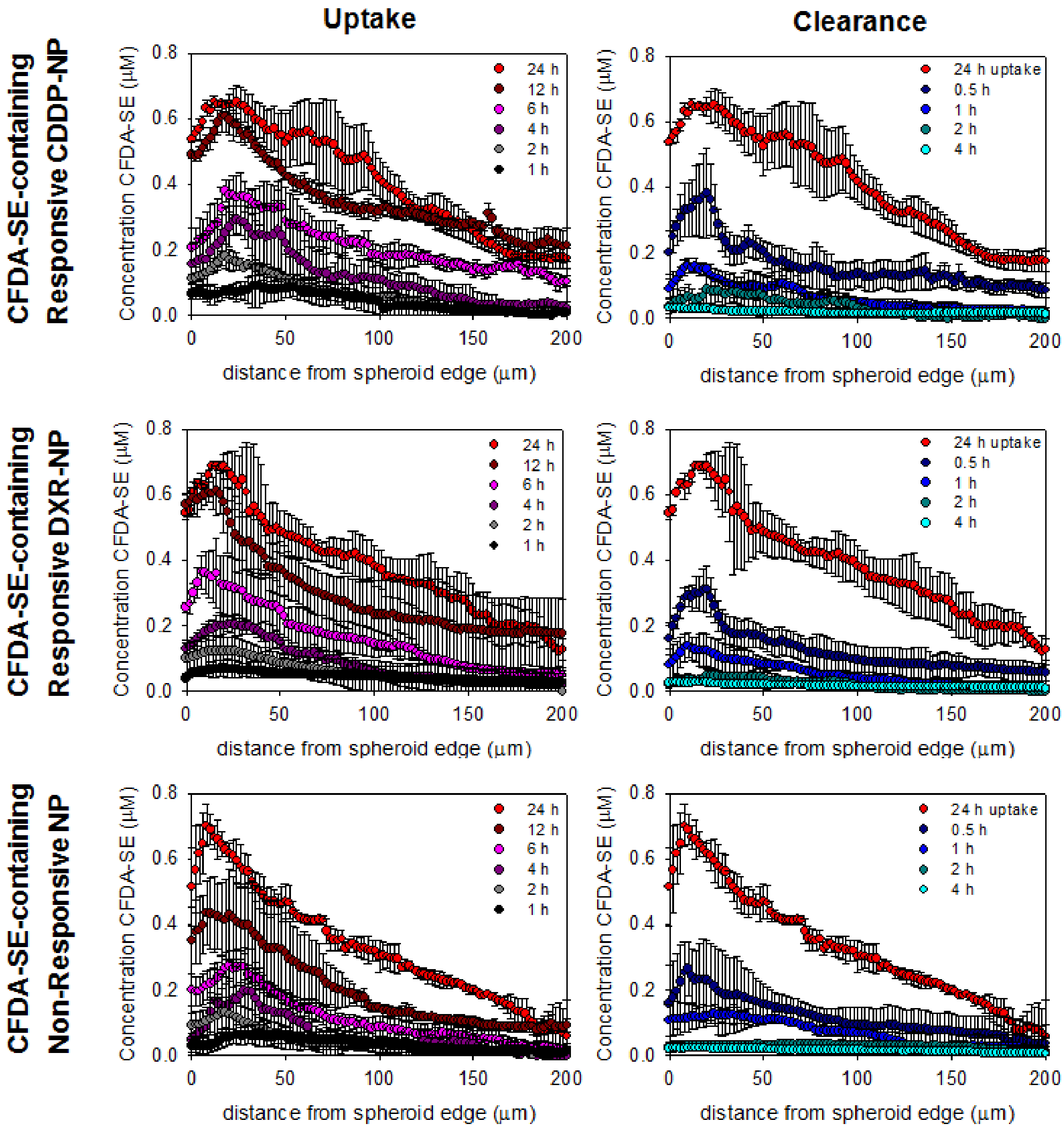

Figure S12. Spatiotemporal microdistributions of the fluorescent drug surrogate CFDA-SE on MDA-MB-231 spheroids delivered by **responsive-NP, designed for CDDP and for DXR, and of non-responsive NP.** All NP were loaded with CFDA-SE. For the 'uptake' microdistributions, spheroids were fished, frozen, and sliced during incubation with 0.8µM CFDA-SE encapsulated in the corresponding NP type (2mM lipid) after 1 (black), 2 (grey), 4 (dark purple), 6 (pink), 12

(maroon), or 24 hours (red symbols) of incubation. For the ‘clearance’ microdistributions, all spheroids were first transferred into fresh media, were fished after 0.5 (navy blue), 1 (royal blue), 2 (green), or 4 hours (cyan), and were then frozen and sliced. Points correspond to the average and standard deviation of n=3 different equatorial sections (from 3 different spheroids) per time point.

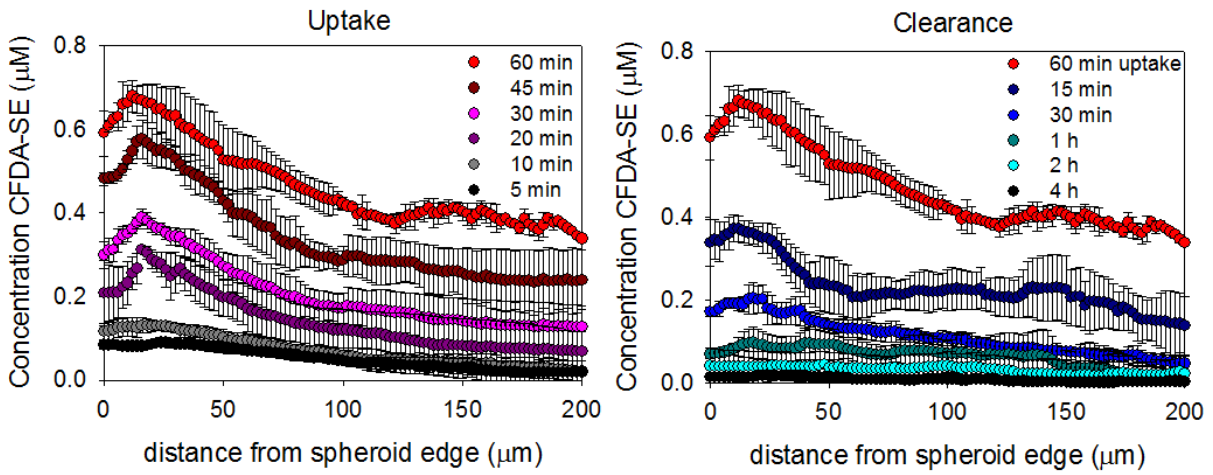

Figure S13. Spatiotemporal microdistributions of the **free fluorophore CFDA-SE**, used as **surrogate of the free chemotherapeutic agents**, on MDA-MB-231 spheroids. For the 'uptake' microdistributions, spheroids were fished, frozen, and sliced during incubation with 0.8μM CFDA-SE after 5 (black), 10 (grey), 20 (dark purple), 30 (pink), 45 (maroon), or 60 minutes (red symbols) of incubation. For the 'clearance' microdistributions, all spheroids were first transferred into fresh media, were fished after 15 minutes (navy blue), 30 minutes (royal blue), 1 hour (green), 2 hours (cyan), or 4 hours (black symbols), and were then frozen and sliced. Points correspond to the average and standard deviation of n=3 different equatorial sections (from 3 different spheroids) per time point.

References

1. Sempkowski, M., et al., *Sticky Patches on Lipid Nanoparticles Enable the Selective Targeting and Killing of Untargetable Cancer Cells.* Langmuir, 2016. **32**(33): p. 8329-38.
2. Stras, S., et al., *Growth of Metastatic Triple-Negative Breast Cancer Is Inhibited by Deep Tumor-Penetrating and Slow Tumor-Clearing Chemotherapy: The Case of Tumor-Adhering Liposomes with Interstitial Drug Release.* Molecular Pharmaceutics, 2020. **17**(1): p. 118-131.
